# Exploring allomelanin: A comparative analysis via natural product extraction and synthesis

**DOI:** 10.1126/sciadv.ady4848

**Published:** 2026-02-13

**Authors:** Sofia Aman, Lauren M. Irie, Shengyi Su, Zofia E. Siwicka, James J. La Clair, Brianna N. Kalaj, Elisabeth I. Latawiec, Prachi Karanjkar, Michael R. Wasielewski, Ali Dhinojwala, Michael D. Burkart, Omar K. Farha, Nathan C. Gianneschi

**Affiliations:** ^1^ Department of Materials Science and Engineering, International Institute for Nanotechnology, Chemistry of Life Processes Institute, Northwestern University, Evanston, IL 60208, USA.; ^2^ Department of Chemistry, International Institute for Nanotechnology, Chemistry of Life Processes Institute, Northwestern University, Evanston, IL 60208, USA.; ^3^ Department of Chemistry and Biochemistry, University of California, San Diego, 9500 Gilman Drive, La Jolla, CA 92093-0358 USA.; ^4^ Department of Chemistry and Institute for Quantum Information Research and Engineering, Northwestern University, Evanston, IL 60208, USA.; ^5^ School of Polymer Science and Polymer Engineering, The University of Akron, Akron, OH 44325, USA.; ^6^ Paula M. Trienens Institute for Sustainability and Energy, Northwestern University, 2145 Sheridan Road, Evanston, Illinois 60208, United States.; ^7^ Department of Chemical & Biological Engineering, Northwestern University, Evanston, Illinois 60208, United States.; ^8^ Department of Biomedical Engineering and Department of Pharmacology, Northwestern University, Evanston, IL 60208, USA.

## Abstract

Allomelanin is a nitrogen-free class of melanin commonly found in plants and fungi. Although synthetic analogs have been developed from 1,8-dihydroxynaphthalene (1,8-DHN), detailed physicochemical comparisons with natural allomelanins remain limited. Herein, we extracted allomelanin from black knot fungus, chaga mushroom, and black oat using an acid-base extraction protocol, comparing them against a library of synthetic analogs derived from a range of putative, natural precursors. Spectroscopic analyses indicate that simple homopolymerization of 1,8-DHN does not adequately represent natural allomelanin structures. Instead, heterogeneous copolymerization of 1,8-DHN with catechol or tannic acid yields materials with physicochemical properties more consistent with natural extracts. This is also supported by their enhanced antioxidant and dye/metal adsorption properties. Like their synthetic counterparts, extracted natural allomelanins exhibit intrinsic porosity, reaching a Brunauer-Emmet-Teller area of 155 square meters per gram, potentially facilitating nutrient transport and toxin adsorption, although further studies will be required to probe this.

## Teaser

Comparing natural and synthetic allomelanin uncovers copolymerization as key to melanin structure and bioinspired material design.

## INTRODUCTION

Melanin, a ubiquitous biopolymer, is broadly classified as a highly cross-linked, heterogeneous polyphenolic (*1*) material with properties including broadband optical absorption, ultraviolet (UV) protection (*2*), and antioxidant capabilities (*3*). These properties have enabled organisms, ranging from animals and plants to fungi and microbes, to adapt to environmental changes such as high radiation (*4*), oxidative stress (*5*), and low nutrient availability (*6*).

Several different classes of melanin have been identified, including eumelanin, pheomelanin, pyomelanin, neuromelanin, and allomelanin (*1*). Among these, the most studied melanin is the nitrogen-containing eumelanin, synthesized from l-3,4-dihydroxyphenylalanine (l-dopa) and related tyrosine-derived precursors (*7*). A lesser studied class of melanin is the nitrogen-free analog, allomelanin, commonly found in plants and fungi (*8*). A wide variety of plant and fungal species with prominent melanized features have been identified. This includes the discovery of high allomelanin content in organisms that exhibit particularly impressive radiation resistance, found in extreme environments such as the Chernobyl Exclusion Zone and the International Space Station (ISS) (*4*, *9*, *10*).

The prevalence of allomelanized organisms in such extreme environments has prompted an interest in synthetic mimics of allomelanin. The initial focus has been on 1,8-dihydroxynaphthalene (1,8-DHN) as a monomer precursor (*11*, *12*). These 1,8-DHN–based synthetic allomelanins were found to exhibit excellent radical scavenging, UV-protective properties, adhesion, tunable hydrophilicity/hydrophobicity, and intrinsic, stable organic radical content (*11*). Furthermore, 1,8-DHN–based allomelanin nanoparticles exhibit intrinsic microporosity, with BET areas as high as 800 m^2^/g, making them an example of a biological polymer of intrinsic microporosity (BioPIM) (*12*). This is in contrast to eumelanin systems, which have been shown to have no substantial porosity (*13*).

These findings motivate the study presented herein, in which we asked two questions. The first question is: Can we validate that naturally occurring allomelanins are BioPIMs? The second question is: Are the chemical identities of natural allomelanin consistent with those of the 1,8-DHN allomelanin analog, or are there significant differences? If so, how do these affect the physicochemical properties observed? In this work, we aim to elucidate the similarities and differences between natural and synthetic systems to simultaneously inform the design of melanin mimetic materials for translational applications and to deepen our understanding of the natural products themselves. In addition, this work prompts the question of the significance of BioPIM presence in these organisms in contrast to other types of melanins that are nonporous and the general advantages of having a high surface area material.

It has been reasoned that extracellular allomelanin may be formed from an amalgamation of canonical allomelanin precursors in addition to other naturally occurring microbial phenols, plant phenols, or agrochemicals that exist in the organisms’ native environment (*14*). We hypothesize that a closer synthetic mimic of natural allomelanins can be achieved by strategically copolymerizing naturally occurring phenolic compounds and comparing them against natural allomelanins to elucidate their chemical properties and functions in relation to each other.

## RESULTS

### Selection of allomelanin from fungi- and plant-based sources

**Fig. 1.**
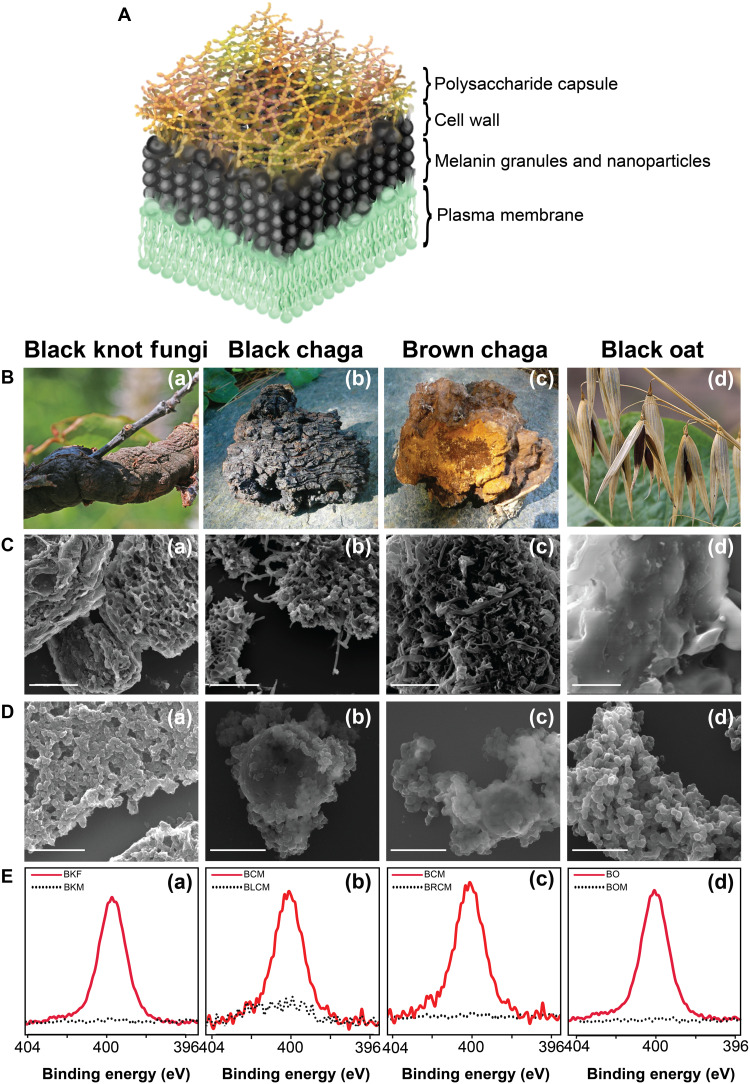
Extraction and characterization of natural allomelanin. (**A**) Model showing the distribution of melanin within the cell wall. (**B**) Natural sources selected for this study. SEM micrographs for ground sources, (**C**) nonextracted (scale bars, 5 μm), and (**D**) extracted melanins (scale bars, 1 μm). (**E**) Nitrogen XPS of nonextracted and extracted melanins. (a) Ground BKF and the associated extracted melanin, BKM. (b) Ground chaga mushroom (BCM) and the associated extracted melanin from the black portion, BLCM. (c) Ground brown chaga mushroom (BCM) and the extracted melanin, BRCM. (d) Ground BO and extracted melanin, BOM. Fungi and oat images are adapted from the literature [(*66*), CC BY 2.0 (https://creativecommons.org/licenses/by/2.0/); (*67*, *68*), CC BY-SA 3.0 (https://creativecommons.org/licenses/by-sa/3.0/deed.en)].

We examined melanin extracted from three species: black knot fungus (BKF), black chaga mushroom (BCM), and black oat (BO) (Fig. 1 and table S1). These sources were selected because of previously established extraction methods in the literature, producing high yields of melanin, as well as being commercially available and readily acquired (*15*–*17*). Furthermore, these species are biologically unique from each other, with distinct native environments, localization of melanin, and pathogenic nature or lack thereof.

BKF or *Apiosporina morbosa* [Fig. 1B(a)] is a pathogenic plant disease native to North America that infects the branches and trunks of plum, cherry, apricot, and chokeberry trees (*15*, *18*). The fungus first penetrates the bark of the host tree, inducing a tumorlike outgrowth, which matures over time to form a woody, swollen, black knot around the infected bark. Dhinojwala and co-workers demonstrated that the pathogenic BKF can be used as a sustainable source of allomelanin (*15*). It was concluded that within the fungus, melanin is strongly conjugated to polysaccharides, namely, chitin, which must be removed through an acid-base extraction method (*15*). This acid-base extraction method can produce a high yield (10% yield from dry mass) of natural allomelanin from its starting material. For comparison and consistency within this study, we replicated the extraction and characterization procedures on BKFs in a similar manner to a previously published work (*15*).

Chaga mushroom, or *Inonotus obliquus* is another parasitic fungal species that grows primarily on birch trees and has been historically used in traditional medicine across Europe and Asia (*19*). These fungi have been used since the 16th century for a wide range of sicknesses, including tuberculosis, heart disease, cancer, skin diseases, and stomach diseases (*20*). These fungi have a high content of melanin in both their black exterior [Fig. 1B(b)] and lighter brown-colored interior [Fig. 1B(c)], which have been isolated via water-based extraction (*21*) and acid-base extractions (*16*). A previous study has also speculated that the melanin extracted from BCM is nitrogen-free allomelanin derived from the 1,8-DHN monomer precursor (*19*).

BO or *Avena strigosa* Schreb. [Fig. 1B(d)] is a cereal crop that belongs to the genus *Avena*. The oat grain has a hull and kernel, where the hull comprises about one-quarter of the whole grain. Other oat species lack the color variation found in BO hulls, which are primarily black and have been identified by researchers as melanized (*17*). Furthermore, a study has shown that the pigment extracted from the oat hull belongs to the family of allomelanin (*17*). This study argued that the precursor for this oat melanin is *p*-coumaric acid, a phenolic compound commonly found in fruits, vegetables, and cereals (*22*).

### Isolation of melanin from natural sources via acid-base extraction

Natural sources of allomelanins were granulated before acid-base extraction using a ball mill, as shown in the Supplementary Materials (fig. S1). We refer to these ground, powdered samples as BKF, BCM, and BO. An acid-base extraction procedure (fig. S1) was used because of its previously reported success in the removal of chitin and proteins associated with melanin found in fungal cell walls (Fig. 1A) (*14*). In this manner, black knot melanin (BKM) was extracted from BKF, and black oat melanin (BOM) was extracted from BO. For the chaga mushroom, black chaga melanin (BLCM) was extracted from the black, hard exterior of the mushroom [Fig. 1B(b)], and brown chaga melanin (BRCM) was extracted from the brown, spongy interior of the mushroom [Fig. 1B(c)]. Once isolated, the natural melanins were characterized to determine the success of the extraction method. First, scanning electron microscopy (SEM) images were taken of the granulated natural samples [Fig. 1C(a to d)] and of the acid-base extracted melanin from these samples [Fig. 1D(a to d)]. The acid-base extraction procedure changed the macroscale morphology of the granulates, transforming the material from large aggregates into smaller, irregularly shaped particulates. To further confirm successful extraction, x-ray photoelectron spectroscopy (XPS) was performed to determine the elemental composition of the preextracted samples (fig. S2A and table S2) and postextracted melanins, BKM, BLCM, BRCM, and BOM (fig. S2B and table S3). The carbon XPS spectra for all extracted melanins have the five different peaks commonly seen in the literature for melanin species (fig. S3 and tables S4 and S5) (*23*, *24*). The nitrogen XPS spectra [Fig. 1E(a to d)] shows the absence of a nitrogen peak postextraction, which is consistent with a nitrogen-free allomelanin material. This strongly suggests that the protein and polysaccharide network surrounding the melanin was successfully removed. These results also highlight that the extracted allomelanins are distinct from eumelanins, demonstrated by the absence of nitrogen peaks (Fig. 1E) and their different carbon XPS spectra relative to nitrogen-rich sepia (eu)melanin (melanin commonly associated with squid inks) (fig. S4) (*15*).

In addition to the acid-base extraction method, a previously reported chemoenzymatic extraction method for eumelanin from mammalian tissues was also tested on the BKF sample (*25*). However, the chemoenzymatic extraction was not as successful in removing the nitrogen-containing polymer (i.e., chitin), as indicated by the nitrogen content in the XPS analysis postextraction, as well as lack of any morphological change in comparison (fig. S5). Moreover, saprobic fungi, including BKF [Fig. 1B(a)], are typically collected (cut and ground) with tissues from their plant-based hosts, which also would require the addition of enzymes to digest plant-based nitrogen-containing polymers such as lignin. Given the success of the acid-base method, further tailoring of a chemoenzymatic approach was not explored.

### Synthetic allomelanin analogs

**Fig. 2.**
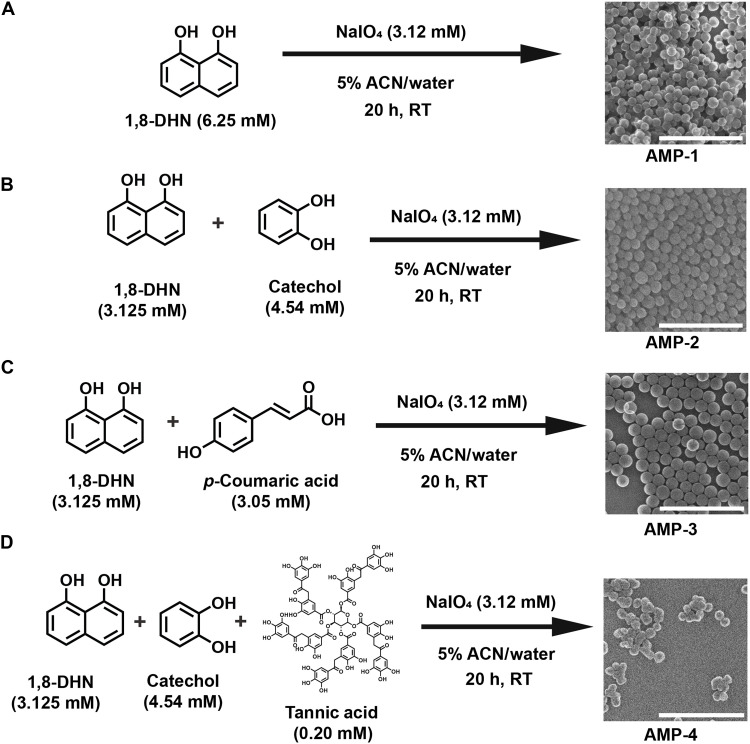
Synthesis of synthetic allomelanin analogs. Scanning transmission electron microscopy-secondary electron (STEM-SE) micrographs of the four synthetic AMP analogs used in this study (**A**) AMP-1, (**B**) AMP-2, (**C**) AMP-3, and (**D**) AMP-4. Scale bars, 1 μm. h, hours; RT, room temperature.

To provide appropriate chemical comparison points against the natural, extracted allomelanin sources, we prepared a series of synthetic allomelanin analogs with varying monomer compositions (Fig. 2). We reasoned that natural melanin likely polymerizes or associates with other naturally occurring phenolic or catecholic molecules (*26*). For example, catechol is a precursor to catechol melanin, a subclass of allomelanin found commonly in the cell wall or spores of a variety of fungal species such as *Ustilago maydis* (*26*). Furthermore, catechol is commonly used in and isolated from a variety of plants and trees (*27*–*29*). In addition, *p*-coumaric acid is a phenolic molecule that has been shown to be a primary constituent of oat melanin and its widespread occurrence in nature (*17*, *22*). Similarly, tannic acid is another phenolic molecule commonly excreted by trees and plants as part of the immune response associated with oxidative stress (*30*, *31*). Because of the natural relevance and accessibility of these molecules, we strategically incorporated them as comonomers with 1,8-DHN.

We designed four types of synthetic allomelanin particle (AMP) analogs. AMP-1 is the result of a homopolymerization of the canonical 1,8-DHN monomer using a synthesis scheme previously reported in the literature (*11*), which forms well-defined spherical nanoparticles (Fig. 2A). AMP-2 is the result of a copolymerization between 1,8-DHN and catechol in a ratio of 1:1 by mass (Fig. 2B). AMP-3 was prepared via the copolymerization of 1,8-DHN and *p*-coumaric acid in a ratio of 1:1 by mass (Fig. 2C). Last, AMP-4 is derived from the copolymerization of 1,8-DHN and catechol in a ratio of 1:1 by mass in the presence of a 0.2 mM tannic acid solution (Fig. 2D). All synthetic allomelanin variations yielded well-defined spherical nanoparticles with dark pigmentation. Furthermore, XPS spectra of all these synthetic melanins showed carbon peaks characteristic of allomelanin (fig. S6 and tables S4 to S6). These synthetic allomelanins were then characterized and compared to extracted allomelanin analogs.

### Understanding the physicochemical nature of isolated and synthetic allomelanin

A comparative study was conducted using two solid-state chemical characterization methods: Fourier transform infrared spectroscopy (FTIR) and solid-state nuclear magnetic resonance (ssNMR). These methods were used to confirm the efficiency of the extraction method (figs. S7 and S8) and to identify similar functional groups across the extracted and synthetic allomelanins.

**Fig. 3.**
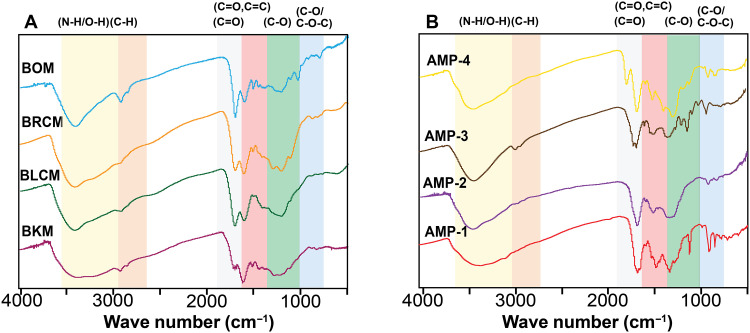
FTIR spectra. (**A**) Extracted melanin. (**B**) Synthetic melanin. These spectra were taken under transmission mode, in solid state using KBr pellets. Both the designed and extracted melanin show similar environments except for a sharp alkyl peak that is present in all the natural melanin and AMP-4.

The FTIR spectra of the fungal sources before extraction have a C─O stretch at 1034, 1069, and 1100 cm^−1^ and a C─O─C stretch at 1155 cm^−1^, associated with the polysaccharides in the cell wall (fig. S7) (*15*, *32*). As expected, these peaks are significantly reduced in the extracted melanin. Crucially, natural melanin samples (Fig. 3A) and all four synthetic allomelanins (Fig. 3B) contained key moieties including C═C and C═O, which are associated with peaks at 1610 and 1660 cm^−1^, O─H between 3600 and 3200 cm^−1^, and the C─O stretch between 1200 and 1400 cm^−1^ (fig. S7D). However, there is a distinct C═O functional group peak found at 1700 cm^−1^ in all natural samples, which is only observed for AMP-4 among the synthetic analogs.

To further probe chemical similarities between the synthetic and natural allomelanins, ^13^C cross-polarization/magic angle spinning ssNMR (^13^C CP/MAS ssNMR) spectra were collected (figs. S8 and S9). Notably, most of the polysaccharides present in all of the powdered allomelanin sources were removed post–acid-base extraction, evidenced by the disappearance of peaks between 50 and 100 parts per million (ppm) (fig. S8, A to D). However, the natural allomelanin spectra still show aliphatic species between 0 and 60 ppm, which is consistent with the previous literature (fig. S9A) (*15*, *33*). ^13^C CP/MAS ssNMR spectra were also collected for all synthetic allomelanin samples (fig. S9B). The AMP-1 ^13^C CP/MAS ssNMR spectrum was similar to that previously reported in the literature (*11*), with peaks corresponding to 1,8-DHN. Although there are similarities in peak shifts between ^13^C CP/MAS ssNMR spectra, direct comparisons between the natural and synthetic allomelanin spectra cannot easily be made because this type of ssNMR is not quantitative.

To address this, ^13^C multiple CP/MAS ssNMR (^13^C multi-CP/MAS ssNMR) spectra were collected as this method provides a quantitative view of the carbon populations relative to one another across melanin samples (fig. S10). ^13^C multi-CP/MAS ssNMR involves the application of multiple alternating ramped-CP periods and ^1^H spin-lattice relaxation periods to provide quantitative ^13^C ssNMR spectra (*34*). This method has been used and validated across various natural organic materials, including fungal eumelanins, plant matter, and kerogen (*34*–*37*). In agreement with the ^13^C CP/MAS ssNMR, the ^13^C multi-CP/MAS ssNMR spectra also reveal a wide range of aliphatic species between 0 and 60 ppm (fig. S10A), indicating that, although most of the chitin and chitosan are removed via acid-base extraction, lipids remain in the natural samples. Furthermore, previous research speculates that these lipids may be covalently attached to the melanin, thus requiring an alternate method to remove them, although this has not been definitively shown (*38*). Notably, although these aliphatic peaks appear prominent in the ssNMR spectra, they are much less prominent in the aliphatic region of the FTIR spectra (2800 to 3150 cm^−1^), with BOM having the highest intensity aliphatic peaks (Fig. 3A and fig. S10A).

**Fig. 4.**
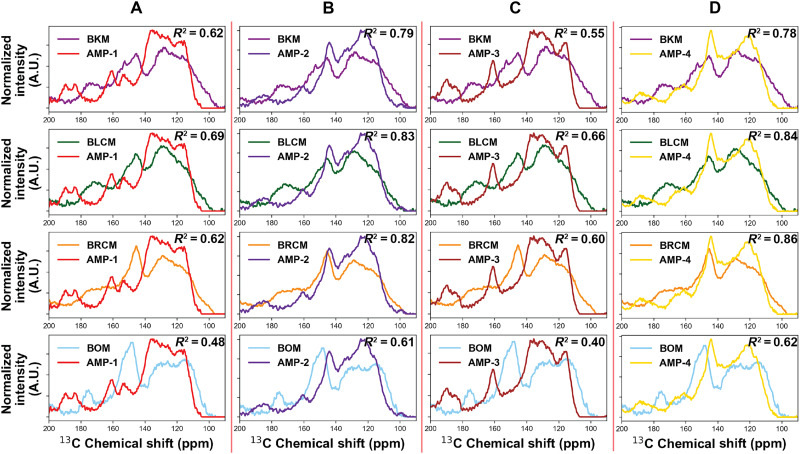
^13^C multi-CP/MAS ssNMR spectra of natural, extracted allomelanin plotted against the developed synthetic allomelanin analogs. (**A**) AMP-1, (**B**) AMP-2, (**C**) AMP-3, and (**D**) AMP-4 multi-CP/MAS ssNMR spectra plotted against those of BKM, BLCM, BRCM, and BOM. A.U., arbitrary units.

Using quantitative ^13^C multi-CP/MAS ssNMR spectra, more direct comparisons between peak shape and population could be made between the natural and synthetic allomelanin analogs. To compare the chemical similarities between the samples, the natural and synthetic allomelanin multi-CP/MAS ssNMR spectra were normalized and plotted against each other between 90 and 200 ppm (Fig. 4). The natural allomelanin spectra (BKM, BLCM, BRCM, and BOM) exhibited three main features including a prominent ─C═CH associated resonance between 120 and 130 ppm, a smaller shoulder between 110 and 120 ppm, as well as a peak at 140 to 150 ppm corresponding to C─OH (*33*, *39*, *40*). A quantitative assessment of the chemical similarities between the natural and synthetic allomelanins was achieved by calculating the correlation coefficient (*R*^2^) between the normalized ^13^C multi-CP/MAS ssNMR spectra (Fig. 4 and fig. S11). AMP-1 and AMP-3 were similar to each other but exhibited dissimilar ^13^C multi-CP/MAS ssNMR spectra from the natural allomelanins, in particular lacking the prominent peak between 140 and 150 ppm. By contrast, the AMP-2 and AMP-4 spectra both featured the C─OH peak between 140 and 150 ppm and, overall, have much more similar spectra to the natural samples in comparison to AMP-1 and AMP-3. *R*^2^ values also support that AMP-2 and AMP-4 spectra are more similar to the various natural allomelanins in comparison to AMP-1 and AMP-3 (Fig. 4, B and D, and fig. S11). Specifically, AMP-4 exhibits the closest resemblance to the two allomelanins derived from chaga mushroom (BRCM and BLCM) indicated by the *R*^2^ value of 0.84 and 0.86, respectively. Both AMP-2 and AMP-4 demonstrate the most similarity to the spectra of BKM and BOM, as indicated by their higher *R*^2^ values. Notably, the spectra of the extracted natural allomelanins are similar to each other, indicated by their *R*^2^ values approaching 1 (fig. S11). This consistency suggests that the carbon chemical signatures of natural allomelanins maintain a high degree of uniformity across diverse biological sources.

A previous study has suggested that the harsh acid-base extraction conditions commonly used for melanin isolation can alter the pigment’s structure, potentially complicating our understanding of its native structure (*41*). To address this, we tested modified extraction protocols using milder acid-base extraction conditions (table S7). Melanin obtained using these conditions exhibited particulate morphologies comparable to those extracted under harsher conditions (fig. S12). The FTIR spectra likewise showed no evidence of additional structural moieties (fig. S13). Similarly, the ^13^C multi-CP/MAS spectra revealed comparable chemical environments across both extraction protocols (fig. S14). However, in contrast to the broad and diffuse resonances typically observed with harsher conditions, the milder extractions yielded narrower peaks. This suggests that, although the chemical environments of melanin are largely preserved irrespective of extraction severity, the milder protocol fails to fully remove surrounding impurities.

As a way to further evaluate the potential effect of the acid-base extraction procedure on melanin’s physicochemical structure, we treated all synthetic allomelanin samples with the acid reflux procedure and their pre- and post-^13^C multi-CP/MAS ssNMR spectra were evaluated. The acid reflux treatment seems to induce changes in the ^13^C multi-CP/MAS ssNMR spectra in AMP-1 and AMP-3, whereas little changes are observed in AMP-2 and AMP-4 (fig. S15, A and B). Ultimately, whereas the acid-treated AMP-1 and AMP-3 did increase the *R*^2^ correlation value with the natural allomelanins in some cases, the acid-treated AMP-2 and AMP-4 still consistently showed higher *R*^2^ correlation values with the natural allomelanins (fig. S15C). The acid reflux–treated AMP-1 was also further characterized, and we observed that, although its FTIR signal was reduced posttreatment, functional groups were not altered, and its particle morphology remained unchanged (fig. S16, A and B). Because the synthetic allomelanin samples showed little change to their physicochemical properties posttreatment with the harsh acid reflux, we speculate that the natural allomelanin samples would also undergo little change during the extraction procedure.

Melanin exhibits a characteristic broadband UV-vis absorption spectra (*42*), which is observed in all the allomelanin analogs used in this study (fig. S17, A and B). Furthermore, the thermal stability of the natural and synthetic allomelanins were also compared by thermogravimetric analysis (TGA). TGA showed that AMP-1 is more thermally stable than AMP-2 and AMP-3, which are both subsequently more stable than AMP-4 and the extracted melanins (fig. S18). This trend demonstrates that increasing the heterogeneity of the material through the inclusion of more distinct monomers leads to a decrease in thermal stability, as supported by the literature (*43*).

**Fig. 5.**
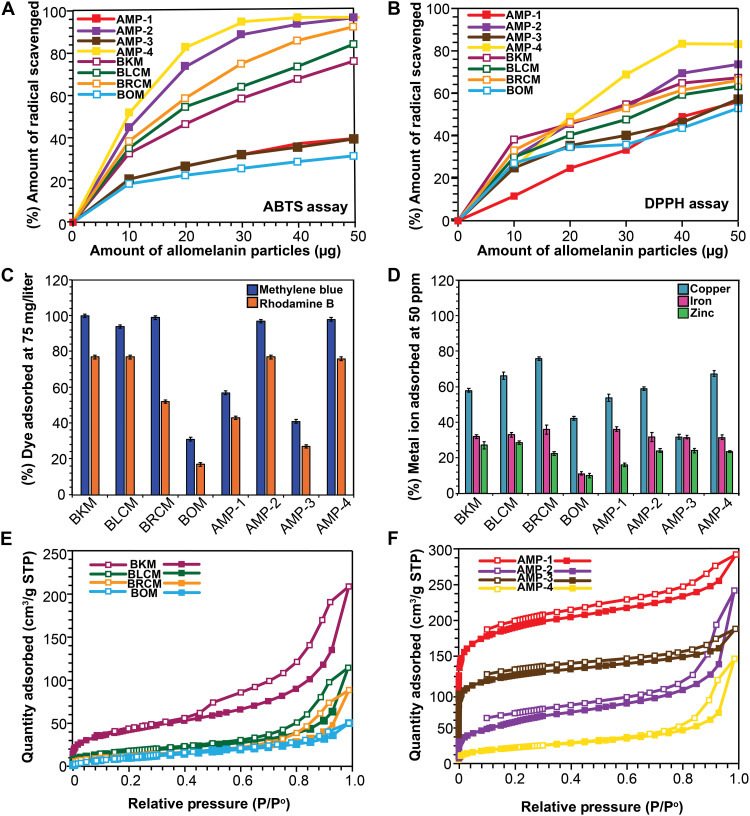
Physical properties of synthetic and natural allomelanin. UV-vis spectra of (**A**) natural allomelanin and (**B**) synthetic allomelanin. (**C**) DPPH radical scavenging assay at 0.2 mM DPPH with 0 to 50 μg of melanin nanoparticles added. (**D**) ABTS radical scavenging assay at 0.2 mM ABTS with 0 to 50 μg of melanin nanoparticles. N_2_ BET isotherms of (**E**) natural allomelanins and (**F**) synthetic allomelanins. STP, standard temperature and pressure.

High antioxidant activity is a hallmark property of melanin extracted from various fungi and plants (*44*). This radical scavenging activity is often attributed to its high free radical content and reversible redox properties (*3*, *45*, *46*). Electron paramagnetic resonance (EPR) analysis of the extracted natural melanin (fig. S19) and synthetic analogs (fig. S20) confirms the presence of free radicals, with *g*-factor values ranging from 2.0036 to 2.0038, consistent with previously reported literature values (table S8). To further investigate the antioxidant properties, two radical scavenging assays were performed using 2,2-diphenyl-1-picrylhydrazyl (DPPH) (*44*) (Fig. 5A) and 2,2′-azino-bis(3-ethylbenzothiazoline-6-sulfonic acid) (ABTS) (Fig. 5B) (*45*). On the basis of these assays, extracted melanin samples have better radical scavenging capabilities compared to the homopolymer AMP-1, except BOM. BOM, AMP-1, and AMP-3 scavenge up to a maximum of 40% at a concentration of 25 μg/ml of particles in the DPPH solution. However, AMP-2, AMP-4, BKM, BRCM, and BLCM not only outperform but can also reach almost twice the percentage of radicals scavenged compared to AMP-1. To understand the influence of monomer ratio on the chemical properties, we synthesized two more allomelanin variations, AMP-5 and AMP-6, where the amount of catechol was reduced to half relative to AMP-2 and AMP-4, respectively. Radical scavenging assays performed on these samples show that antioxidant activity increased with the amount of catechol used in the synthesis (fig. S21).

Melanin also functions as a natural small-molecule adsorber and metal chelator (*47*). To evaluate these properties, both natural and synthetic allomelanins were incubated in solutions containing different cationic dyes (methylene blue and Rhodamine B) (Fig. 5C) and various metal ions (Fig. 5D). Among the natural samples, BOM exhibited the lowest dye adsorption capacity, comparable to AMP-1 and AMP-3, whereas AMP-2 and AMP-4 showed performance similar to BKM, BLCM, and BRCM. In contrast, metal ion adsorption displayed selectivity. For example, although all samples demonstrated the highest chelation affinity for copper ions, BOM consistently underperformed. Notably, AMP-2 and AMP-4 matched the adsorption capacities of the natural samples for both copper and zinc. Iron adsorption, however, remained highly consistent across all samples, underscoring melanin’s dual role as both a reservoir and a sink for specific metal ions (*48*).

### Intrinsic porosity of allomelanin

It has been previously established that AMP-1 exhibits intrinsic microporosity and small-molecule adsorption capabilities (*12*). Thus, nitrogen isotherm measurements for all allomelanin samples were taken (Fig. 5, E and F) to determine their BET areas, pore volumes, and DFT-calculated pore size distributions (fig. S22 and table S9).

For synthetic allomelanin nanoparticles, AMP-1 exhibited the highest surface area at 800 m^2^/g, similar to the previously reported literature (*12*). Copolymerization of 1,8-DHN with the phenolic molecules led to significant decreases in BET area. AMP-2 had a lower BET area of 205 m^2^/g. AMP-3 exhibited a BET area of 450 m^2^/g. AMP-4 had the lowest BET area among synthetic allomelanins at 79 m^2^/g. For the natural allomelanins, BKM exhibited the highest porosity with a BET area of 155 m^2^/g, a pore size distribution including micropores at 1.4 nm, and a wide range of mesopores. BLCM and BRCM also exhibited non-negligible surface areas of 50 and 60 m^2^/g, respectively. BOM showed the lowest but non-negligible surface area of 38 m^2^/g. Notably, of all the natural allomelanins, BKM was the only sample that showed a high N_2_ uptake at low pressure. This initial high uptake is also exhibited in AMP-1, AMP-2, and AMP-3 but not AMP-4.

N_2_ isotherms were performed on both the mild acid-base extracted and chemoenzymatically extracted black knot samples. Both these conditions failed to effectively degrade and remove the complex biopolymers found in the hard fungi, including but not limited to peptides and chitin impurities from the melanin samples (figs. S16 and S23), rendering the resulting melanin extracts nonporous (fig. S24 and tables S10 and S11). This finding raised questions about whether the porosity seen in natural BKM melanin is merely a by-product of the acid-base extraction process. To investigate this, a nitrogen isotherm measurement was carried out on synthetic AMP-1 melanin, both before and after acid-base extraction conditions were applied. The porosity remained consistent and unchanged across these tests (Fig. 5F and fig. S16C), indicating that the observed porosity in natural melanin samples is not an artifact. It is possible that the apparent porosity of the natural melanins is not intrinsic rather induced by the melanin growth on a template of biomacromolecules that are removed during the acid-base extraction, leaving behind a porous architecture. Although this phenomenon may account for some of the macro- or mesoporous features of the natural allomelanins, the presence of micropores in many of the natural allomelanins are too small to be formed by biomolecules that typically exist as 20- to 30-nm bundles within plant and fungal cell wall (*32*, *49*). Furthermore, the lower BET areas measured for the natural, extracted samples, in comparison to the synthetic ones, can likely be attributed to the incomplete removal of excess lipids, as discussed in previous sections. These findings underscore the need for extraction methods tailored to the biological source and intended application as no single protocol is likely to be universally applicable.

## DISCUSSION

### Allomelanin: A heterogeneous biopolymer

Our objective was to compare and contrast the leading synthetic allomelanin analog in the literature (AMP-1) against extracted natural allomelanins. In addition, a small library of alternative synthetic allomelanin analogs (AMP-2, AMP-3, AMP-4) was achieved through spontaneous incorporation of phenolic molecules into 1,8-DHN–based synthetic allomelanins via oxidative polymerization. This study shows that extracted natural allomelanins are more physicochemically similar to the heterogeneous synthetic allomelanin analogs, AMP-2 and AMP-4, when comparing their ^13^C multi-CP/MAS ssNMR and FTIR spectra as well as their radical scavenging properties and metal and dye remediation behavior.

As discussed previously, the native fungal environment is rich in naturally occurring phenolic and catechol-rich molecules. What sets fungal allomelanins apart from other types of melanin is their extracellular localization in the cell wall, where they act as a robust defensive layer that interfaces with the external environment (*6*). We speculate that perhaps these melanized fungi adeptly incorporate these phenolic/catecholic compounds into their biological architecture, thereby conserving resources and leveraging their surroundings. From a perspective of survival and the inherent characteristics of allomelanin, it is not entirely unexpected that allomelanin exhibits the capacity to assimilate materials from its environment, a concept alluded to in the existing scientific literature (*26*, *50*). Furthermore, these typically parasitic fungal species, such as BKFs and chaga mushrooms, depend on their host trees for nutrients. Many of these trees often release substances such as tannic acid to manage immune responses or alleviate oxidative stress (*30*, *31*). We speculate that allomelanin within parasitic fungi might sequester such secreted antagonistic compounds from their hosts to fortify themselves. This adsorptive behavior of allomelanin is also supported by a previous work, which shows that synthetic allomelanins can be used to sequester small molecules such as pesticides and chemical warfare agents (*12*).

This study further demonstrates that the integration of phenolic/catecholic molecules into allomelanin alters its properties including porosity and radical scavenging activity. Notably, of all the samples studied, the AMP-1 homopolymer exhibits the highest surface area, as measured by BET analysis. However, when other monomers were copolymerized with 1,8-DHN to prepare analogs (AMP-2, AMP-3, and AMP-4) there was a decrease in BET surface area, aligning more closely with the BET surface area of natural allomelanins. Intriguingly, despite its high surface area, AMP-1 displays only moderate radical scavenging activity and dye/metal adsorption compared to the natural allomelanins like BKM, BLCM, and BRCM and synthetic allomelanins like AMP-2 and AMP-4. These findings suggest that, in this case, radical scavenging activity is influenced more by chemical composition than by BET surface area alone. The incorporation of catechol (AMP-2) and catechol/tannic acid (AMP-4) significantly enhances radical scavenging activity, reaching levels comparable to BKM, BRCM, and BLCM. This indicates that the inclusion of these monomers induces chemical modifications that translate into improved antioxidant behavior. Notably, BOM, a naturally sourced allomelanin from a noninvasive plant species (*17*), exhibits moderate radical scavenging and adsorption capacities similar to AMP-1 and AMP-3, suggesting underlying chemical or morphological differences between BOM and other natural allomelanin sources. Along the same lines, chemical composition rather than BET surface area also seems to affect its dye and metal adsorption properties as AMP-2 and AMP-4 had much more similar activity to BKM, BRCM, and BLCM compared to AMP-1.

Overall, these results show that we can tune and optimize the properties of synthetic melanin analogs through the copolymerization of the 1,8-DHN monomer with these catecholic/phenolic molecules. This ability to tune radical scavenging activity can have large implications in applying these materials to applications such as in therapeutics or radiation protection, where scavenging reactive oxygen species or other free radicals is imperative. Similarly, tuning the dye and metal adsorption capabilities of synthetic melanins can also be beneficial for remediation applications.

### Biological significance of porosity in allomelanin

Melanin in nature is located both extracellularly and intercellularly in the cell wall, depending on the fungal or plant species (*51*). Within the cell wall, allomelanin provides structural support (*52*), regulating cell permeability and turgor forces (*53*), and providing protection from xenobiotics and toxins (*6*). The discovery of intrinsic microporosity within the natural allomelanin samples is especially intriguing because eumelanin, including sepia-derived and synthetic eumelanin analogs such as polydopamine nanoparticles, does not exhibit porosity (*13*). More precisely, no known extracted natural eumelanin sources exhibit intrinsic microporosity (*54*). We elaborate previously that heterogeneity of natural allomelanins can enhance the antioxidant activity and dye/metal adsorption of the resulting material. The porosity of these nanomaterials, which also results from this heterogeneous chemical composition, suggests that there are evolutionary advantages to porosity as well.

Previous studies have shown that nonmelanized cell walls are more permeable toward antifungals (e.g., amphotericin B and caspofungin) due to the presence of large pores in the cell wall (*55*, *56*). In contrast, melanized fungal species exhibit a reduced susceptibility to toxic metal ions such as silver (*57*). We speculate that this resistance may be due to the evolution of porous melanins that reduce the diffusion of toxins through adsorption and adhesion while still facilitating nutrient transport. Some support for this theory comes from studies on antifungal susceptibility assays on melanin and nonmelanin-expressing strains (*58*). This theory is supported by observations of certain melanized pathogenic fungi that are less susceptible to antifungals (*59*), an immunity that could be partially attributed to the porosity of the melanin present (table S9).

In addition, high surface areas would increase interactions with other biomolecules such as lipids and polysaccharides. This work revealed that, once extraneous polysaccharides and proteins are removed from the BKFs, the extracted allomelanin exhibits significant porosity and an increased BET area (Fig. 5D and table S9). We propose that this intrinsic microporosity enhances the interfacial interactions between melanin and other molecules, which can be highly beneficial for the organisms. For example, a greater porosity may lead to higher loading of lipids, which leads to increased energy storage and fungal virulence, aiding in survival (*60*). High surface area melanins facilitate an inherent increase in interactions with structural macromolecules like chitin, which can enhance the mechanical strength of the cell wall. There is precedence in the literature that synthetic melanin nanomaterials improve the mechanical properties of polymer composites (*61*, *62*).

It is established that both nutritional and environmental elements influence pigment production across species (*51*). Melanin offers protection against multiple harmful environmental factors, ranging from high radiation, oxidative damage, and metal toxins. It also supports effective thermal regulation (*1*, *63*). Although we speculate that allomelanin’s microporosity contributes favorably to the cell wall by providing structure while allowing permeation of nutrients and ions, further investigation into the fungi’s adaptive capabilities and the extent of their melanization is required to better understand the role allomelanin plays in plants and fungi (*8*, *51*).

In summary, through extracting melanin from multiple sources and characterizing their physicochemical properties, we show that two of the strategically designed synthetic analogs, AMP-2 and AMP-4, show much more similarities with the natural allomelanin samples in contrast to AMP-1. These results imply that the homopolymerization of 1,8-DHN is not necessarily representative of natural allomelanin—rather, a heterogeneous mixture of 1,8-DHN with other naturally occurring phenolic monomers like catechol and tannic acid may be spontaneously incorporated into the resulting melanin material. We show that the inclusion of such monomers can introduce enhanced radical scavenging and dye/metal adsorption properties in the synthetic melanins, more closely mimicking those of natural allomelanins. Furthermore, we conclude that some allomelanins in nature exist as BioPIMs. We speculate that this porosity could be advantageous for the cell wall, lending adhesion, maintaining mechanical integrity, and providing fungi and plants with a route to allow nutrient transportation as well as metal chelation, preventing toxin exposure. However, this hypothesis requires further exploration to better understand the role of nitrogen-free melanins across natural systems.

## MATERIALS AND METHODS

### Experimental design

#### Acid-base extraction of melanin

Melanin-rich areas were scraped off to isolate the powdered initial nonextracted samples BKF and BCM, and for BO, the husks were separated from the oat. The separated parts of the fungi and oats were then crushed into fine powder using a ball mill at room temperature for 10 min. After which, the collected powder was boiled in water before being treated with a solution of 1 M NaOH. The NaOH solution was allowed to sit for 30 min to allow for maximum solubilization of melanin in the base. Afterward, the solution was autoclaved, followed by centrifugation, and the supernatant was collected. The supernatant was treated with concentrated HCl, allowing the solution to reach a pH of 1. This allows the melanin to precipitate out of the supernatant, which is then collected via centrifugation. Last, to allow any residual proteins and lipids to hydrolyze, the precipitate was acid refluxed at 120°C for 20 to 24 hours and recollected after centrifugation and three water washes and one ethanol wash. The significance of each step is discussed in the Supplementary Materials. Additional modifications to the method were implemented to extract melanin from natural sources under gentler acid and base treatment conditions, as described in detail in the Supplementary Materials (Methods and table S7).

#### Chemoenzymatic extraction of melanin

This process was conducted in a 15-ml (sample volumes of 10 ml) or 50-ml (sample volumes of >10 ml) Falcon tube (Fisher Scientific), and all incubations were stirred by inversion on a rotisserie tube rotator. Centrifugation was conducted on MultifugeX3R (Fisher Scientific).

The process involves five enzymatic treatments followed by a chemical gravimetric purification (*25*). Briefly, a sample of a crude BKF (500 mg) was suspended in 10 ml of phosphate buffer (PB; pH 7.2) and treated with 166 mg of cellulase (50 U, 300 U/mg) from *Aspergillus niger* (C1184, MilliporeSigma) for 48 hours at 37°C. The mixture was centrifuged at 4200 rpm and washed with PB (pH 7.2; 2 × 10 ml). Next, the resulting powder was resuspended in 10 ml of PB (pH 7.2) and treated with 250 mg (50 U, 200 U/mg) chitinase from *Streptomyces griseus* (C6137, MilliporeSigma) for 48 hours at room temperature. The mixture was centrifuged at 4200 rpm and washed with PB (pH 7.2; 2 × 10 ml). The resulting powder was resuspended in 10 ml of PB (pH 7.2) and treated with 50 mg (50 U, 1000 U/mg) β-glucanase from *Trichoderma longibrachiatum* (G4423, MilliporeSigma) for 48 hours at 37°C. The mixture was centrifuged at 4200 rpm and washed with PB (pH 7.2; 2 × 10 ml). After completion of carbohydrate digestion, steps were taken to clear oligonucleotides and lipids from this material. Each sample was resuspended in H_2_O (25 ml) and 1.5 mg of deoxyribonuclease I (3000 U, 2000 U/g) from the bovine pancreas (D4527, MilliporeSigma). The sample was sonicated for 5 min (3-s on, 4-s off). The resulting suspension was allowed to precipitate by gravity for 12 hours. The supernatant was removed. The sample was washed three times with water and centrifugation (4200 rpm). This treatment served to remove oligonucleotides from the material. The final enzymatic step was used to remove lipid contaminants. The resulting powder was suspended in H_2_O (25 ml) and 1.5 mg of Amano lipase (30 U, 20,000 U/g) from *Pseudomonas fluorescens* (534730, MilliporeSigma). The sample was sonicated for 5 min (3-s on, 4-s off), and the suspension was allowed to precipitate by gravity. The supernatant was removed, and the sample was washed with water (2 × 10 ml). The resulting powder was transferred to a glass vial (20 ml) and suspended in MeOH (8 ml), and CH_2_Cl_2_ (4 ml) was added until the black powder settled to the bottom. This procedure was repeated three times. The resulting powder was dried under a vacuum to afford 185 mg of black powder.

#### Synthesis of synthetic allomelanin analogs

All allomelanin analogs were polymerized through oxidative polymerization using sodium periodate. For AMP-1, 1,8-DHN (150 mg, 6.25 mM) was dissolved in 150 ml of water solution containing 5% acetonitrile (ACN), followed by an addition of 1 ml of 0.47 M sodium periodate (100.15 mg, 3.12 mM). AMNP-2 was prepared by dissolving 1,8-DHN (75 mg, 3.125 mM) and catechol (75 mg, 4.54 mM) in 150 ml of water containing 5% ACN, followed by an addition of 1 ml of 0.47 M sodium periodate (100.15 mg, 3.12 mM). AMNP-3 was prepared by dissolving 1,8-DHN (75 mg, 3.125 mM) and *p*-coumaric acid (75 mg, 3.05 mM) in 150 ml of water containing 5% ACN, followed by the addition of 1 ml of 0.47 M sodium periodate (100.15 mg, 3.12 mM). AMNP-3 was prepared by dissolving 1,8-DHN (75 mg, 3.125 mM), catechol (75 mg, 4.54 mM), and tannic acid (50 mg, 0.2 mM) in 150 ml of water containing 5% ACN, followed by an addition of 1 ml of 0.47 M sodium periodate (100.15 mg, 3.12 mM). AMP-5 and AMP-6 were synthesized using the same protocol as for AMP-2 and AMP-4, respectively, but the catechol was decreased to 1/2 mass equivalence (37.5 mg, 2.27 mM). For all particles, following the injection of sodium periodate, the reaction mixtures were stirred for 20 hours at room temperature before being centrifuged and washed three times with Milli-Q water.

#### CP/MAS ^13^C solid-state nuclear magnetic resonance

Thirty to 40 mg of the melanin sample was packed into a 4.0-mm MAS rotor from Bruker. The temperature was set to 288 K. Each one-dimensional (1D) ^13^C cross-polarization (CP) was acquired using 20k scans on a 750-MHz ^1^H spectrometer (Bruker). All ^13^C NMR spectra were recorded with complete proton decoupling at 83 kHz. The spinning speed was 15.5 kHz. ^13^C chemical shifts were referenced to adamantane external reference at 38.3 ppm and reported in ppm. Spinning sidebands from the NMR spectra were verified to not overlap with peaks of interest. When there was uncertainty, the CP experiment was run at multiple spinning frequencies to verify peaks. FID files were processed using TopSpin 3.2 (Bruker) and MestreNova 6.0.2 (Mestrelab Research). Peaks were identified in each sample spectra and compared with the literature and ChemDraw of monomers of interest.

#### ^13^C multi-CP/MAS ssNMR

^13^C multi-CP/MAS ssNMR was recorded on a Bruker Advance III 400-MHz spectrometer equipped with a Bruker 4-mm HX MAS probe by following the previous publication (*34*). The π/2-pulse lengths were 2.1 μs for ^1^H and 3.65 μs for ^13^C. The ^1^H decoupling field strength was 119 kHz during signal detection. The MAS rotation frequency (o_r_) is set to 14 kHz, with a t_z_ delay of 1 s (T_1H_ is about 0.5 s, and T_1H_ is the spin-lattice relaxation time of proton), incorporated within the multiple CP ramp, each having a contact time of 0.5 ms. The spectra were recorded with 1024 transients. The recycle delay was set for 1 s. The proton spin relaxation in the rotating frame (T_1pH_) was found around 8000 μs.

Processing and analysis of ^13^C multi-CP/MAS ssNMR were all coded in Python. For both natural and synthetic allomelanin samples, the integration of the spectral region of interest (90 to 200 ppm) was normalized and plotted against each other. To provide a semiquantitative comparison between the natural and synthetic allomelanin spectra, the correlation coefficient (***R***^**2**^) between each of the natural allomelanins (BKM, BLCM, BRCM, and BOM) spectra and each synthetic allomelanin spectra (AMP-1, AMP-2, AMP-3, and AMP-4) within the spectral region of interest was determined. The ***R***^**2**^ value between each of the spectra within the natural allomelanins was also calculated to gauge the chemical similarities between the natural, extracted allomelanin sources. The ***R***^**2**^ value between two spectra was determined using the following equation (Eq. 1)

$$R^{2}=1-\frac{\mathrm{RSS}}{\mathrm{TSS}}=1-\frac{\sum {\left(a_{i}-z_{i}\right)}^{2}}{\sum {\left(a_{i}-\bar{z_{i}}\right)}^{2}}$$ (1)

where *RSS* is the sum of squares of residuals and *TSS* is the total sum of squares, $a_{i}$ is the *y* value at the *x* value, i, of the first spectra and $z_{i}$ is the *y* value at the *x* value, i, of the second spectra, and $\bar{z_{i}}$ is the average of *y* values in the second spectra.

#### Nitrogen sorption measurements

Synthetic allomelanin samples stored in Milli-Q water were centrifuged at 11,500 rpm for 12 min, and the water was removed and replaced with EtOH. This process was repeated two more times with the addition of fresh EtOH each time to ensure an effective removal of water. Samples were activated using a Tousimis SAMDRI-PVT-3D Advanced Manual Critical Point Dryer. Using the supercritical dryer, particles were added to the sample chamber, cooled to 0° to 10°C, and pressurized to 800 psi. EtOH was exchanged with liquid CO_2_ over 10 hours, purging the system for 5 min every 2 hours. After the fifth purge, the temperature was raised to 40°C and the system was pressurized to 1200 to 1400 psi to obtain supercritical CO_2_. The pressure was released slowly overnight at a rate of 0.5 cm^3^/min. Samples were immediately transferred onto a Micromeritics Smart VacPrep and were placed under vacuum for at least 2 hours at 50°C before sorption measurements. For natural allomelanin samples stored in Milli-Q water, water was centrifuged out, and the samples were lyophilized before sorption measurement. Nitrogen physisorption measurements were collected using a Micromeritics ASAP 2420 instrument at 77 K. Pore size distributions were obtained using density functional theory (DFT) calculations with a carbon slit geometry and an N_2_ DFT model.

#### EPR spectroscopy

Samples were added to quartz capillaries (1.50-mm inside diameter, 1.80-mm outer diameter) as water suspensions at equal volumes. Continuous wave EPR spectra were collected at X-band (∼9.6 GHz) on a Bruker ELEXSYS E680 EPR spectrometer with a Bruker 4122 SHQE-W1 resonator. Measurements were performed with a magnetic field modulation amplitude of 2 G, modulation frequency of 100 kHz, and a nonsaturating microwave power of 1.586 mW. The reported spectra are an average of 32 scans. The spectra were baseline corrected and doubly integrated in Origin 2022. To quantify radical content within the samples, a calibration curve was prepared by measuring the EPR of 4-hydroxy-TEMPO at 5, 10, 100, and 500 μM concentrations in water and used to calculate the concentration of free radical content expressed in units of moles of radical per gram of melanin following a previously established protocol (*64*).

All spectral fits were performed in MATLAB with home-written scripts and the simulation package EasySpin v6.0-dev.5411 (*65*), using the garlic function. The parameters for the simulations (the Gaussian linewidth, Lorentzian linewidths, and *g*-factors) are from a best fit of the data using esfit and the Nelder-Mead Simplex algorithm. The TEMPO standards were used to calibrate the *g*-factors.

#### Radical scavenging assays

DPPH and ABTS assays were performed as per the literature. For the DPPH assay, a 0.2 mM stock solution of DPPH was prepared in 95% ethanol solution. Two series of 100-μl solutions of the AMPs in ethanol were added in 2-ml vials, with concentrations varying from 0 to 50 μg. In these 100 μl of AMP solutions, 1.8 ml of DPPH stock solution was added to one series of solutions for the sample absorbance readings and to the other 1.8 ml of 95% ethanol and water was added for the control absorbance readings. The vials were left in the dark for 20 minutes before their absorbance was monitored at 516 nm. For the ABTS assay, the ABTS bulk solution composed of 2.45 mM of potassium persulfate and 7 mM ABTS dissolved in a 10-ml volume and left to sit in the fridge for 16 hours. From that bulk solution, a 0.2 mM ABTS stock solution was prepared. Two series of 100-μl solutions of the AMPs in ethanol were added in 2-ml vials, with concentrations varying from 0 to 50 μg. In these 100 μl of AMP solutions, 1.5 ml of ABTS stock solution were to one series of solutions for the sample absorbance readings and to the other 1.5 ml of water was added for the control absorbance readings. The vials were left in the dark for 20 minutes before their absorbance was monitored at 740 nm. The radical scavenging activity at each AMP concentration was calculated using the following equation (Eq. 2)

$$I=\left[1-\frac{\left(\mathrm{Ai}-\mathrm{Aj}\right)}{\mathrm{Ac}}\right]\times 100\%$$ (2)

where *Ai* is the control absorbance, *Aj* is the sample absorbance, and *Ac* is the radical solution absorbance.

#### Dye and metal adsorption experiments

Dye adsorption assays were conducted using a dye solution (75 mg/liter) of methylene blue and Rhodamine B in ultrapure water. A 500-μg solution of particles was suspended in 1 ml of dye solutions and incubated for 1 hour on the shaker plate at 180 rpm. To determine dye content adsorbed by particles, the dye solution with particles was spun down and supernatants along with just dye solutions were added to a 96-well plate. Absorbances were taken using a plate reader (PerkinElmer 2300 EnSpire Multimode Plate Reader) at specific wavelengths: methylene blue at 664 nm and Rhodamine B at 554 nm.

Metal adsorption studies were conducted using 50 ppm solutions of copper sulfate, iron chloride, and zinc chloride. A total of 2.5 mg of particles was added to 5 ml of each salt solution, yielding a final particle concentration of 0.5 mg/ml. The suspensions were incubated on a shaker plate at 180 rpm for 3 hours, after which the samples were centrifuged, and the supernatant was collected for analysis. One milliliter of the supernatant was then diluted to 10 ml with 3% nitric acid before measurement by inductively coupled plasma optical emission spectrometry (ICP-OES).

### Materials

BKF was obtained from a local farm in Ohio, chaga mushroom chunks were purchased online through CHI Chaga, and BO was obtained from Green Cover. 1,8-DHN was purchased from Matrix Scientific. Sepia melanin, synthetic eumelanin, *p*-coumaric acid, tannic acid, catechol, sodium hydroxide (NaOH), hydrogen chloride (HCl), DPPH, ABTS, sodium periodate (NaIO_4_), high-performance liquid chromatography (HPLC) grade ACN, HPLC grade ethanol, methylene blue, and Rhodamine B were purchased from Thermo Fisher Scientific. Copper sulfate, iron chloride, and zinc chloride salt were acquired from Alfa Aesar. All chemicals were used as received. Ultrapure water was purified using the Barnstead GenPure xCAD Plus system from Thermo Fisher Scientific and was used in all the experiments. Grids for transmission electron microscopy and silica wafers for SEM were purchased from Electron Microscopy Sciences (EMS).

## Supplementary Materials

### This PDF file includes:

Supplementary Methods Instrumentation Figs. S1 to 24 Tables S1 to S11 References
