# Supplementary material for: Exploring allomelanin: A comparative analysis via natural product extraction and synthesis
Source: Sci Adv. 2026 Feb 13;12(7):eady4848. doi: 10.1126/sciadv.ady4848 (PMC12904162; doi:10.1126/sciadv.ady4848)
Supplement: Supplementary file 1 — Supplementary Methods Instrumentation Figs. S1 to 24 Tables S1 to S11 References [file sciadv.ady4848_sm.pdf]

Science Advances
AAAS

Supplementary Materials for

Exploring allomelanin: A comparative analysis via natural product extraction and synthesis

Sofia Aman *et al.*

Corresponding author: Nathan C. Gianneschi, nathan.gianneschi@northwestern.edu

Sci. Adv. **12**, eady4848 (2026)
DOI: 10.1126/sciadv.ady4848

This PDF file includes:

Supplementary Methods
Instrumentation
Figs. S1 to 24
Tables S1 to S11
References

Methods

Acid-Base Extraction of Melanin: Alkaline solutions enable the effective dissolution of melanin from its surrounding cellular environment; therefore, a 1 mol L^{-1} NaOH solution at pH 14 was used to extract melanin from its natural sources. (*15*) Autoclaving further accelerates this process by providing high pressure and temperature, while simultaneously eliminating bacterial contamination commonly present in fungal samples. Despite this, residual impurities often remain, highlighting the need to tailor extraction conditions for each system to ensure the isolation of pure melanin. The final step involves acid hydrolysis, which precipitates the dissolved melanin from the alkaline extract and removes loosely bound impurities such as lipids, fats, and other aliphatic species. While one might consider using acid hydrolysis alone for melanin isolation, this approach is limited by melanin's poor solubility in acidic media and the potential for structural degradation under prolonged, harsh acid conditions, as reported in the literature. (*42*)

Acid Base Extraction of Melanin at Milder Conditions: To assess the impact of acid–base extraction conditions and individual steps, we modified the protocol to employ milder conditions and introduced specific reaction controls. The main changes are summarized in Table S1, but key adjustments included using NaOH at pH 10 instead of pH 14 and adjusting the acid hydrolysis step to precipitate dissolved melanin at pH 2.5 rather than pH 1. Additionally, one batch was processed without autoclaving, and all batches were washed sequentially with different organic solvents: ethanol, acetone, and methanol. These modifications allowed us to evaluate the necessity of each condition and step for effectively separating melanin from surrounding impurities.

Acid Reflux Treatment of Synthetic Allomelanin nanoparticles: Synthetic allomelanin samples were incubated and hydrolyzed in a pH 1 hydrogen chloride solution at 120 °C for 24 hours.

Instrumentation

Scanning Transmission Electron Microscopy (TEM): Dry state TEM of synthetic allomelanin nanoparticles was collected on a Hitachi STEM HD2300 at an accelerating voltage of 200 kV, under SE mode.

Scanning Electron Microscopy (SEM): Pristine and extracted melanin samples were drop-cast on a silica wafer and coated with 18nm of osmium before imaging. The images were acquired on EPIC SEM FEI Quanta 650.

X-ray photoelectron Spectroscopy (XPS): XPS samples were prepared by drop-casting on a silicon substrate and collected on a Thermo Fisher Scientific XPS, NEXSA G2. The data was analyzed using Thermo Scientific Avantage data system software, and spectra were referenced to the C1s peak at 284.8 eV. The carbon XPS spectra were fit using the five peaks mentioned in the literature for melanin samples: C-C/C=C (284.8 eV), C-O/C-N (286.2 eV), C=O (287.8eV), O-C=O (289.1eV) and π-π* (290-291).(*23,24*)

Fourier transform infrared spectroscopy (FTIR): FTIR spectra were obtained on Thermo Nicolet iS50 under transmission mode, in the solid state using KBr pellets.

Thermogravimetric thermal analyses (TGA): The analyses were performed in a TA TGA5500 instrument under a nitrogen atmosphere using Al_2O_3 pans. Samples were heated from 30°C to 1200°C at 10°C/min and then cooled back down to 30°C at a rate of 20°C/min. Automatic baseline corrections were automatically applied within the instrument.

Dynamic Light Scattering and Zeta Potential: Hydrodynamic diameters and Zeta Potentials were measured using a Malvern Instruments Ltd. Nano ZS in ultra-milli-Q water at room temperature.

Ultraviolet Visible Spectroscopy (UV-Vis): UV-Vis spectra were collected in 1M NaOH using a Nanodrop 2000c UV-Vis Spectrophotometer, in the range of 220-750 nm.

List of Figures

Fig. S1. Acid-Base extraction of natural melanin from chosen plant and fungal.

Fig. S2. A) XPS survey of pristine fungal and plant sources, B) XPS survey of acid-base extracted melanin. We observe the complete absence of nitrogen in the extracted melanin, which provides further evidence that the extracted melanin is allomelanin. The sidebands seen in the 200 eV region are due to silica, as the samples were deposited on a silica wafer.

Fig. S3. Carbon XPS spectra for extracted melanin from natural sources, A) Black Knot melanin, B) Black Chaga melanin, C) Brown Chaga melanin, D) Black Oat melanin.

Fig. S4. A) XPS Survey spectra and B) Carbon spectra for Sepia (Eu)melanin. We see predominant C-C-, C-OH/C-N and C=O peaks.

Fig. S5. SEM images and respective XPS spectra of chemoenzymatic extracted natural samples A) BKF, B) BKM-Cellulase, C) BKM- Beta Glue, D) BKM Chitinase. SEM images show no morphological difference between the extracted and pristine sample BKF, which is further emphasized by the presence of Nitrogen in the XPS spectra. Scale bar: 30 µm

Fig. S6. High resolution XPS spectra of C_{1s} for synthetic analogues, A) AMP-1, B) AMP-2, C) AMP-3, D) AMP-4. We see prominent C-C and C-OH peaks and only minor C-O and O-C=O peaks, different from DHI/DHICA-based Eumelanin as shown in Figure S4.

Fig. S7. Comparative FTIR analysis of natural sources and extracted melanin with color-coded key for peak assignments.

Fig. S8. ^{13}C multi-CPMAS ssNMR analysis of pristine sources and extracted melanin. A) BKF, B) BOM, C-D) BCM, and E) Chitin Structure. We observe a broadening associated with the structure of melanin, and a decrease in peaks associated with chitin in the extracted.

Fig. S9. ^{13}C CPMAS ssNMR spectra of A) natural extracted allomelanin (BKM, BLCM, BRCM, BOM), B) synthetic allomelanins (AMP-1, AMP-2, AMP-3, AMP-4).

Fig. S10. Full ^{13}C multi-CPMAS ssNMR of A) natural, extracted allomelanins (BKM, BLCM, BRCM, BOM) and B) synthetic allomelanin analogues (AMP-1, AMP-2, AMP-3, AMP-4).

Fig. S11. Correlation coefficients (R^2) indicating similarities between ^{13}C multi-CPMAS ssNMR spectra of natural and synthetic allomelanins. Note that when comparing natural allomelanin to itself, the R^2 value will be 1.

Fig. S12. SEM extracted of black knot melanin (BKM) using milder acid base conditions as mentioned in Table S7. We see similar particulate morphology for each extraction protocol. Scale bar 2µm.

Fig. S13. FTIR of black knot melanin extracted under conditions following Table S7. Analysis done in solid state using a KBr pellet. IR spectrum showcases no significant changes in the chemical structure of the melanin.

Fig. S14. 13C multi CP-MAS ssNMR evaluation of alternative extraction techniques (Table S7). (A, B, C) Spectra plotted against the control extraction protocol (BKM-A). (D) Correlation coefficients (R^2) indicating similarities between ^{13}C multi-CPMAS ssNMR spectra of different extraction techniques. Note that when comparing natural allomelanin to itself, the R^2 value will be 1.

Fig. S15. ^{13}C multi-CPMAS ssNMR for acid-reflux treated synthetic melanin. (A) ^{13}C multi-CPMAS ssNMR spectra of the acid-treated synthetic melanins and their non-treated counterparts (B) Correlation coefficients ($\boldsymbol{R^2}$) indicating similarities between ^{13}C multi-CPMAS ssNMR spectra of acid-treated synthetic melanins and their non-treated counterparts. (C) Correlation coefficients ($\boldsymbol{R^2}$) indicating similarities between ^{13}C multi-CPMAS ssNMR spectra of acid-treated and non-acid treated synthetic melanins and the acid-base extracted natural allomelanins

Fig. S16. Characterizing acid-treated AMP-1. A) STEM micrograph (scale bar 1μm), B) FTIR, C) Nitrogen Isotherm.

Fig S17. UV-Vis Absorbance for (A) Natural Melanin (B) Synthetic Melanin at 10ug/mL concentration in water.

Fig. S18. Thermogravimetric analysis of all extracted and synthetic allomelanin samples.

Fig.S19. EPR of natural extracted allomelanin samples.

Fig. S20. EPR of synthetic allomelanin.

Fig. S21. Radical Scavenging Assays for synthetic allomelanin variants AMP-5 (3.125mM 1,8-DHN, 2.27mM Catechol) A) ABTS assay, B) DPPH assay, and AMP-6 (3.125mM 1,8-DHN, 2.27mM Catechol, 0.2 mM Tannic Acid) C) ABTS assay and D) DPPH assay.

Fig. S22. DFT calculated pore size distribution for A) synthetic allomelanin B) natural extracted allomelanin from Nitrogen isotherms at 77 K.

Fig. S23. ^{13}C CPMAS ssNMR spectra of enzymatically extracted melanin from BKF. We observe that there was no decrease in the signals of protein and polysaccharides adhered to melanin compared to the pristine Black Knot Fungi.

Fig. S24. N_2 Isotherms at 77 K for extracted Black Knot Melanin (BKM) using (A) Milder acid-base conditions as reported in Table S7, (B) Chemoenzymatic extraction. BKM-A and BKM-B exhibit similar isotherm profiles, indicating that the choice of solvent does not significantly influence the porosity of the samples. However, under both milder acid-base extraction conditions and chemoenzymatic conditions, impurities surrounding the melanin are not effectively removed, leading to a reduction in the observed porosity showcased via negligible uptake.

Table S1. Location of melanin within the selected sources and the natural environments these plants and fungi are found in.

Table S2. Atomic percentages of different elements as detected by XPS for pristine natural sources.

Table S3. Atomic percentages of different elements as detected by XPS for extracted melanin.

Table S4. Binding energy (eV) of different carbon bonds in both synthetic melanin analogues and natural melanin extracts via XPS. All peaks were charge shifted at 284.eV for Carbon.

Table S5. Area percentages of different carbon bonds in synthetic melanin analogues and natural melanin extracts as determined by XPS. The ratio of C–C/C=C to C–O peaks in natural melanin samples (except BKM) is notably lower, likely due to residual covalently bound lipids and reminiscent of chitin and polyscaharides (15) that remain after extraction. This results in a dominant C-O band that masks other carbon signals, altering the relative area percentages. This feature is also seen in FTIR and ssNMR data. Additionally, sample-specific impurities contribute to variations in peak intensities, making this XPS data primarily qualitative. Furthermore, differences among the synthetic melanins are expected, as they are co-polymers composed of various monomer species.

Table S6. Atomic percentages of different elements as detected by XPS for synthetic allomelanin analogues.

Table S7. Acid base extraction of melanin from natural sources at milder modified conditions.

Table S8. Free Radical content, linewidth, and g-factor values for allomelanin as determined by EPR using a 4-hydroxy TEMPO calibration curve, developed freshly on the day of each measurement.(65) The line width of the melanin species is narrower than what is reported in the literature,(45, 46) This is attributed to the fact that we ran our samples in a liquid state which rather than a solid state, leading to a more non-aggregated sample, therefore avoiding the freezing effect.

The reduced g-factor relative to nitrogenous eumelanin analogues indicates free, stable radicals with a predominantly C–C centered character in the fungal melanin and its mimics.(45)

Table S9. Surface characterization and porosity parameters for synthetic and natural melanin samples.

Table S10. Porosity parameters for black knot melanin (BKM) extracted through milder acid-base conditions, as mentioned in Table S7.

Table S11. Porosity parameters for chemoenzymatically extracted melanin from Black Knot Fungi (BKF).

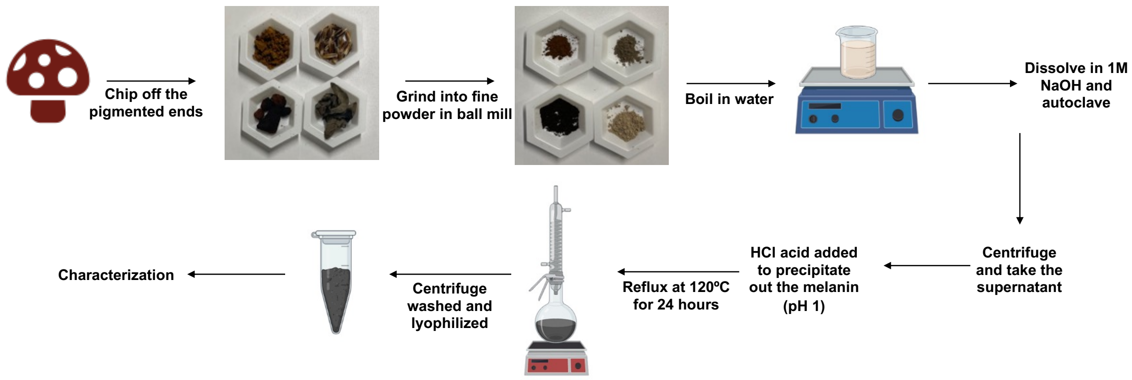

Fig. S1. Acid-Base extraction of natural melanin from chosen plant and fungal sources.

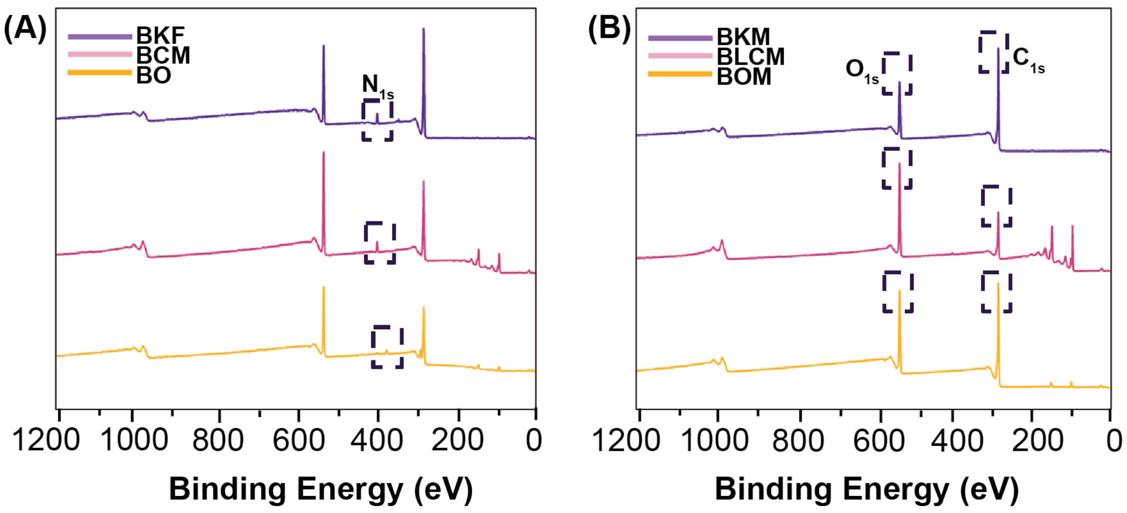

Fig. S2. XPS Survey Spectra A) pristine fungal and plant sources, B) acid–base extracted melanin. We observe the complete absence of nitrogen in the extracted melanin, which provides further evidence that the extracted melanin is allomelanin. The sidebands seen in the 200eV region are due to silica, as the samples were deposited on a silica wafer.

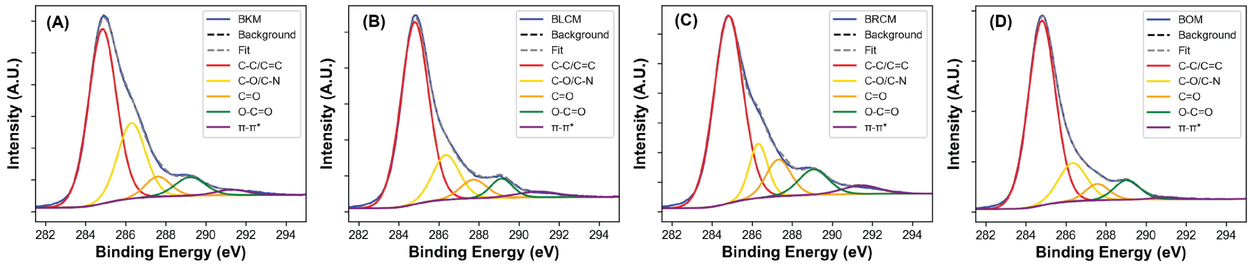

Fig. S3. Carbon XPS spectra for extracted melanin from natural sources, A) Black Knot melanin, B) Black Chaga melanin, C) Brown Chaga melanin, D) Black Oat melanin.

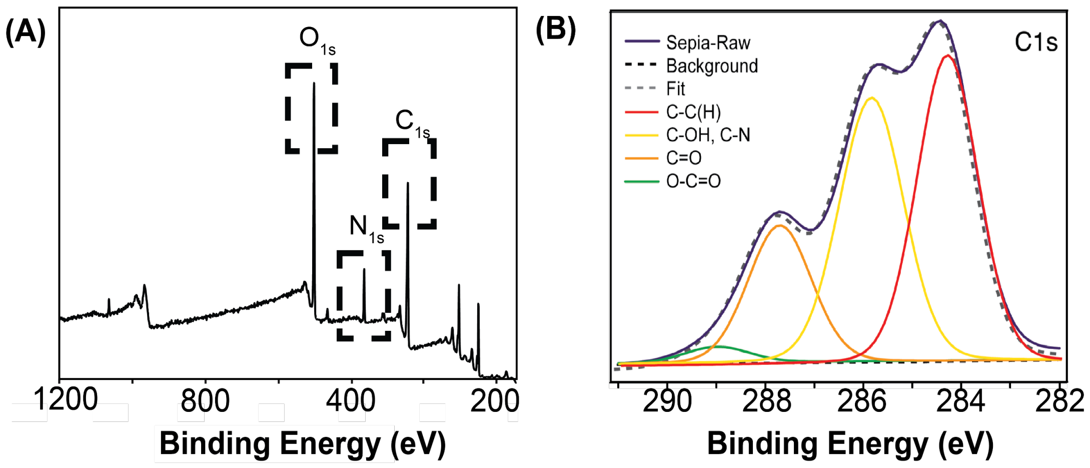

Fig. S4. XPS characterization of Sepia (Eu)melanin, A) Survey spectra and B) Carbon spectra for Sepia (Eu)melanin. We see predominant C-C-, C-OH/C-N and C=O peaks.(*15*)

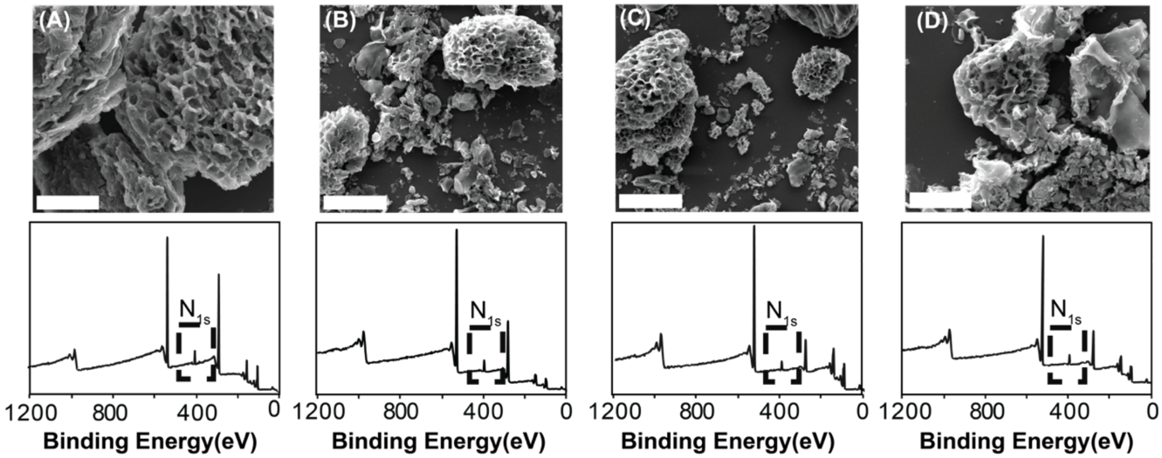

Fig. S5. SEM images and respective XPS spectra of chemoenzymatic extracted natural samples A) BKF, B) BKM-Cellulase, C) BKM- Beta Glue, D) BKM Chitinase. SEM images show no morphological difference between the extracted and pristine sample BKF, which is further emphasized by the presence of Nitrogen in the XPS spectra. Scale bar: 30 μm.

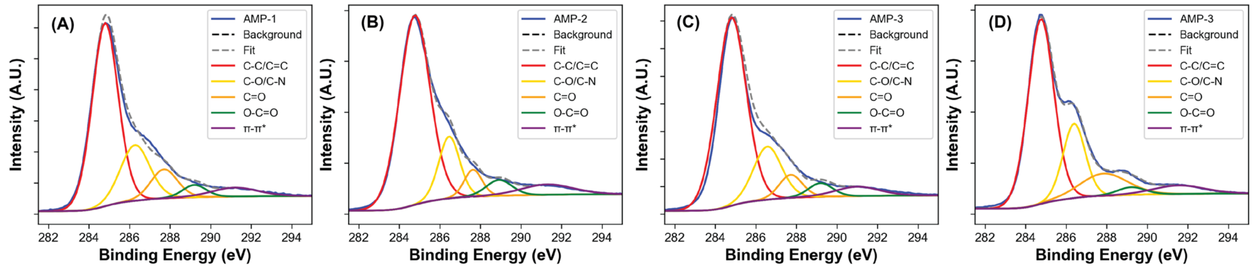

Fig. S6. High resolution XPS spectra of C_{1s} for synthetic analogues, A) AMP-1, B) AMP-2, C) AMP-3, D) AMP-4. We see prominent C-C and C-OH peaks and only minor C-O and O-C=O peaks, different from DHI/DHICA-based Eumelanin as shown in Figure S4.

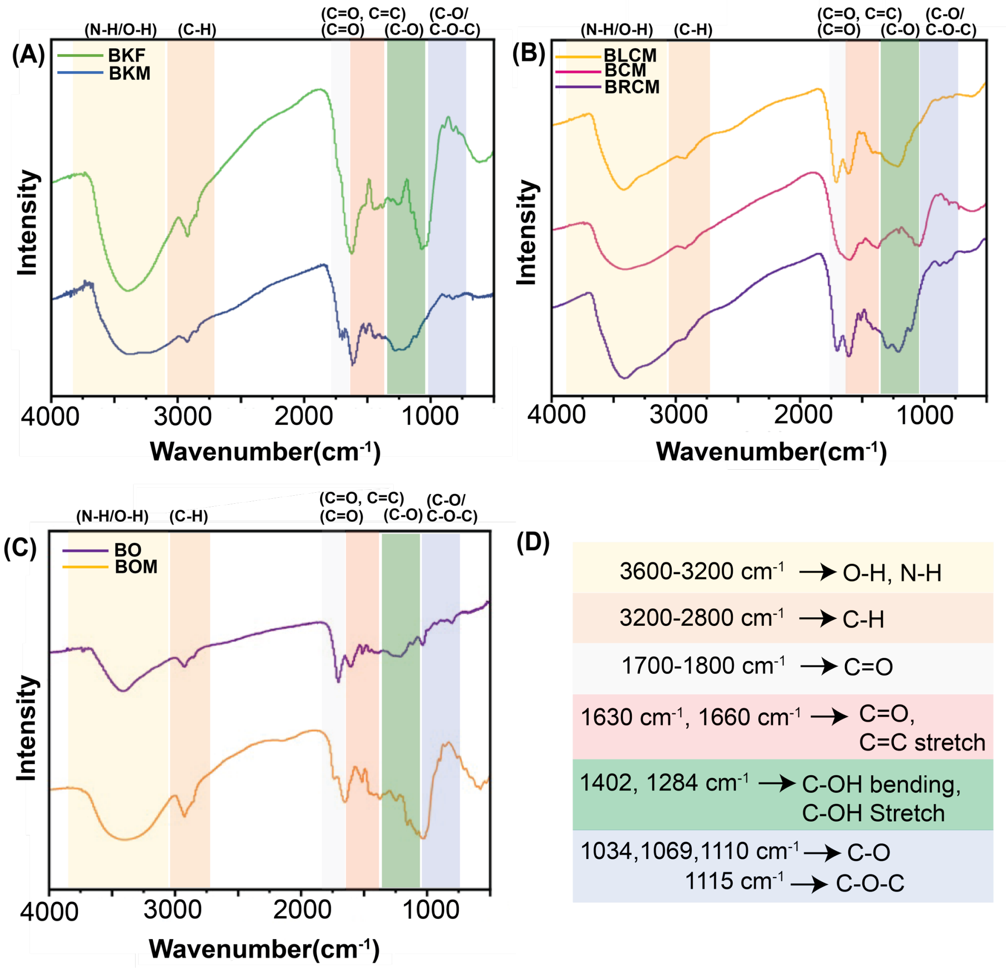

Fig. S7. Comparative FTIR analysis of natural sources and extracted melanin with color-coded key for peak assignments.

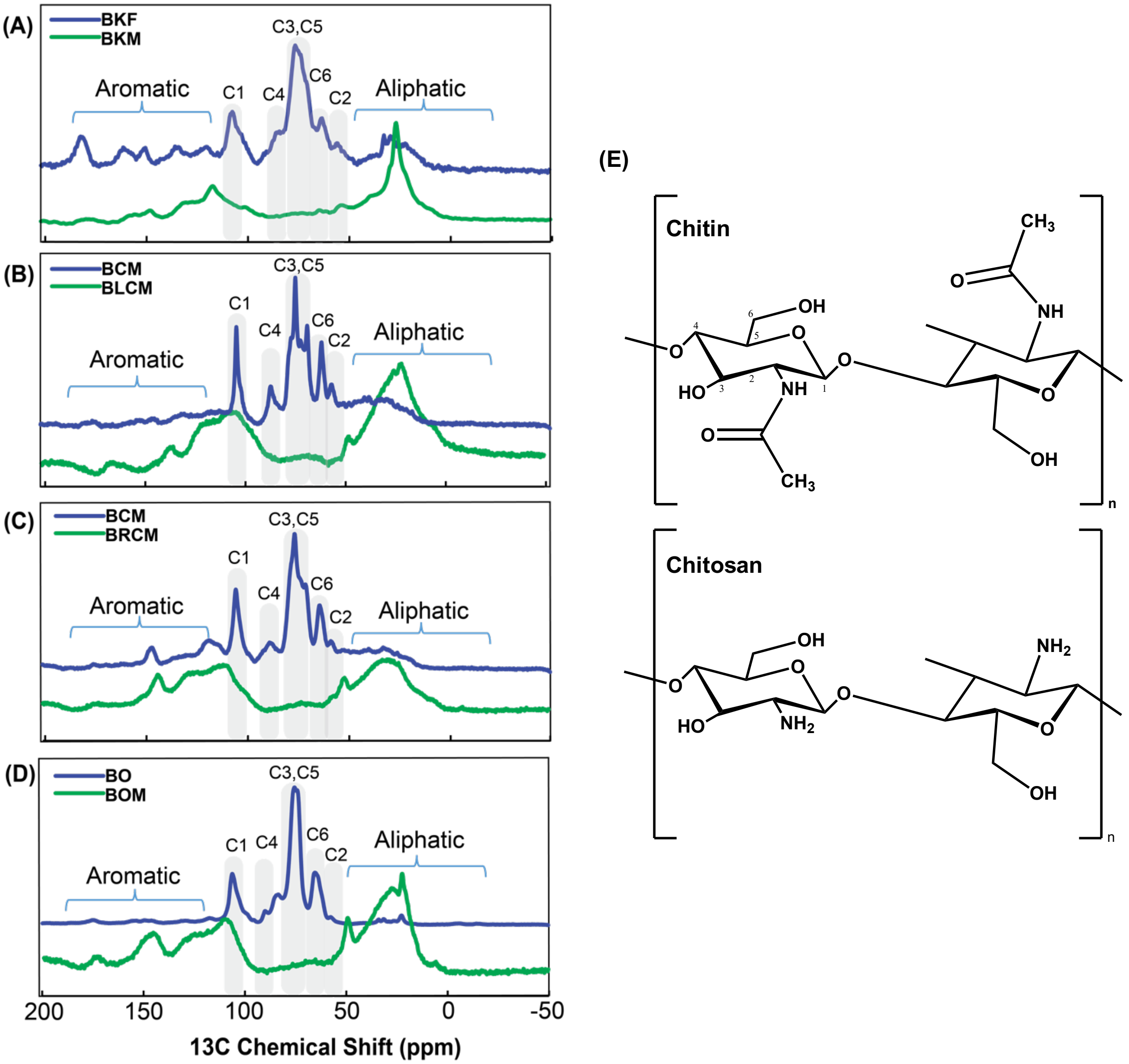

Fig. S8. ^{13}C multi-CPMAS ssNMR analysis of pristine sources and extracted melanin. A) BKF, B) BOM, C-D) BCM, and E) Chemical Structures. We observe a broadening associated with the structure of melanin, and a decrease in peaks associated with the polysaccharides in the extracted.

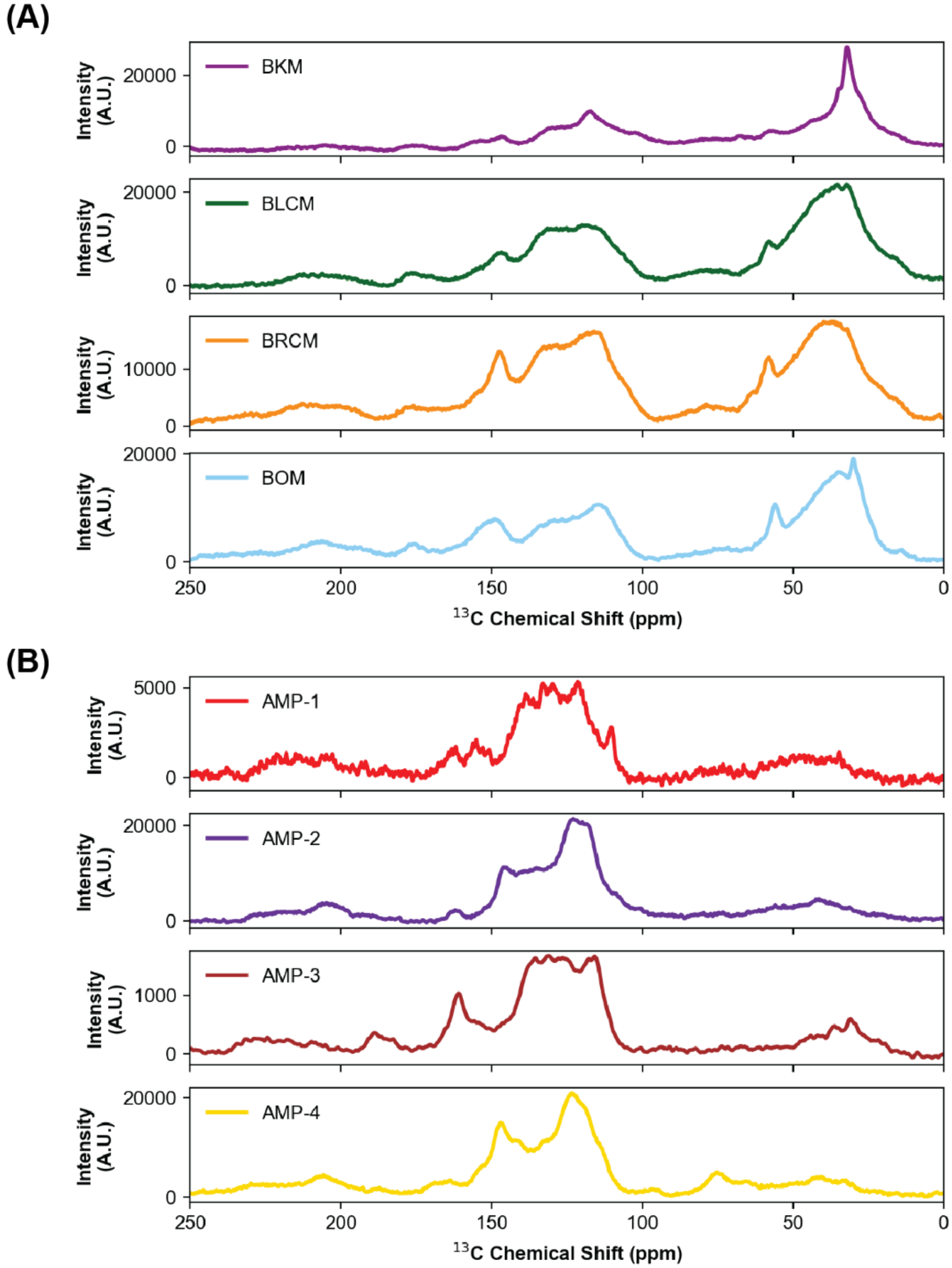

Fig. S9. ^{13}C CPMAS ssNMR spectra of A) natural extracted allomelanin (BKM, BLCM, BRCM, BOM), B) synthetic allomelanins (AMP-1, AMP-2, AMP-3, AMP-4).

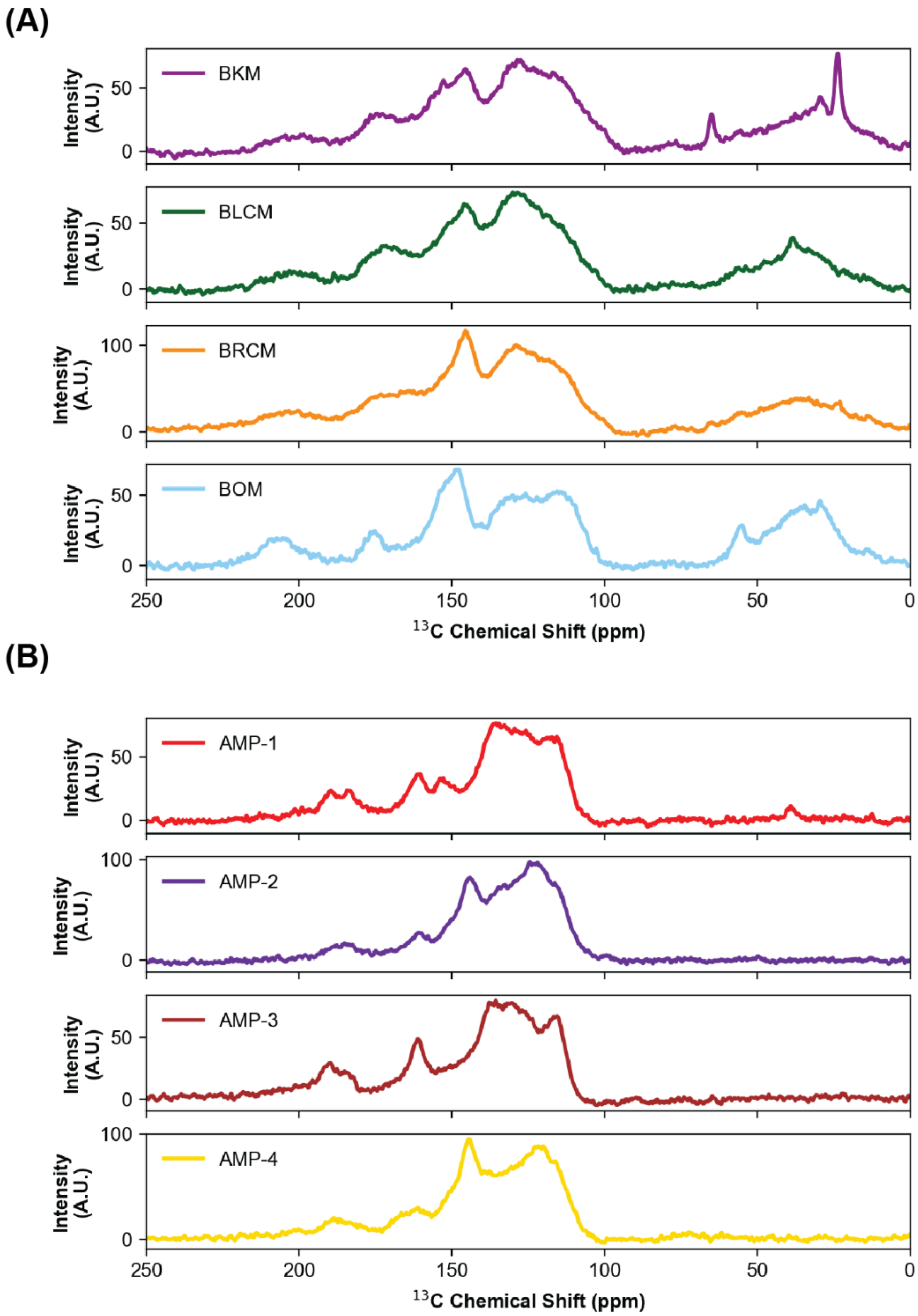

Fig. S10. Full ^{13}C multi-CPMAS ssNMR of A) natural, extracted allomelanins (BKM, BLCM, BRCM, BOM) and B) synthetic allomelanin analogues (AMP-1, AMP-2, AMP-3, AMP-4).

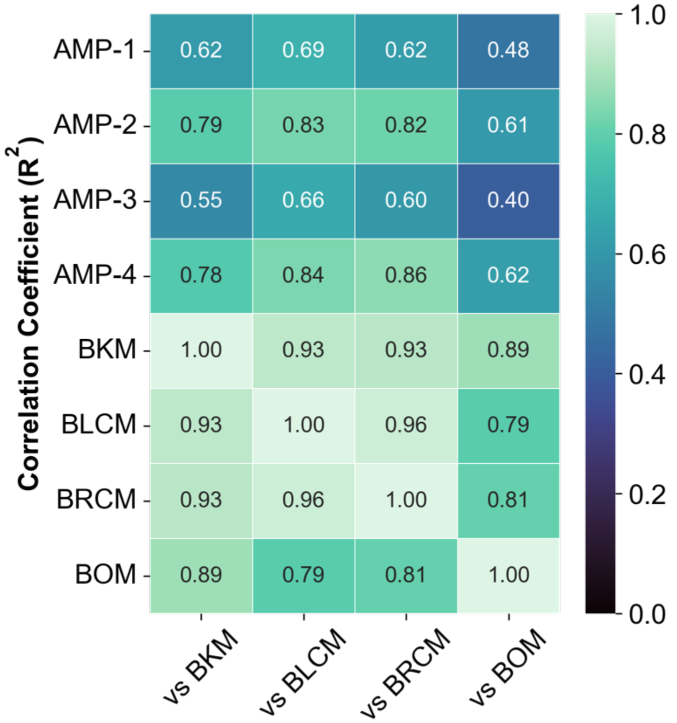

Fig. S11. Correlation coefficients (R^2) indicating similarities between ^{13}C multi-CPMAS ssNMR spectra of natural and synthetic allomelanins. Note that when comparing natural allomelanin to itself, the R^2 value will be 1.

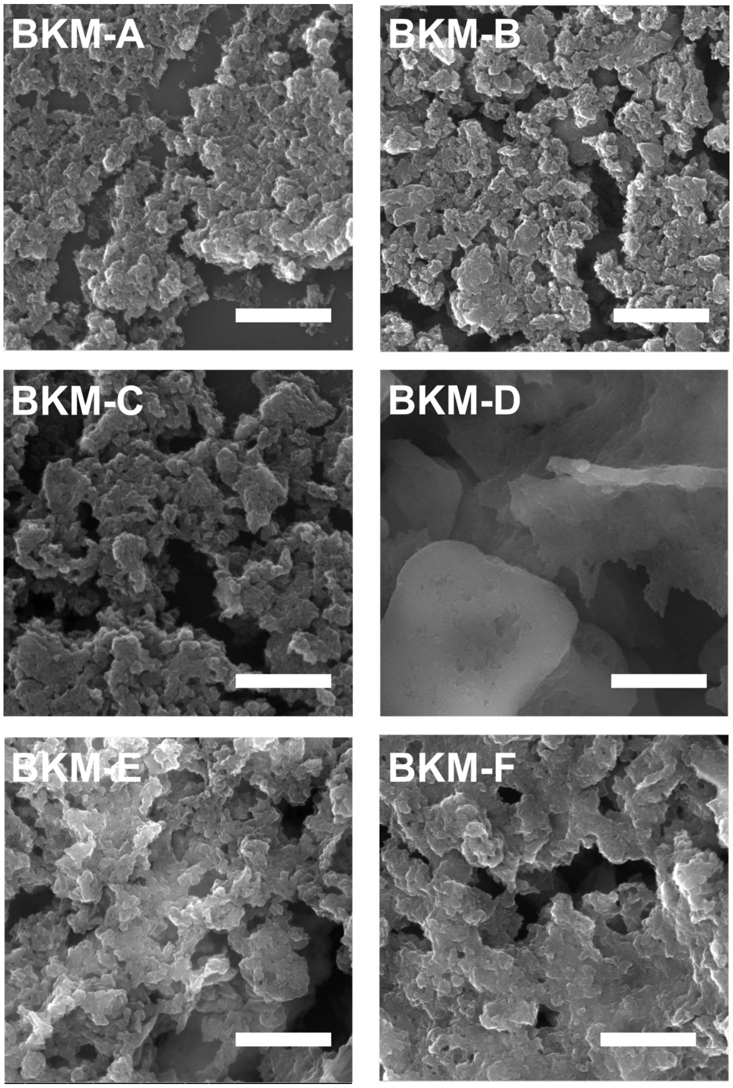

Fig. S12. SEM of extracted black knot melanin (BKM) using milder acid base conditions as mentioned in Table S7. We see similar particulate morphology for each extraction protocol. Scale bar 2μm.

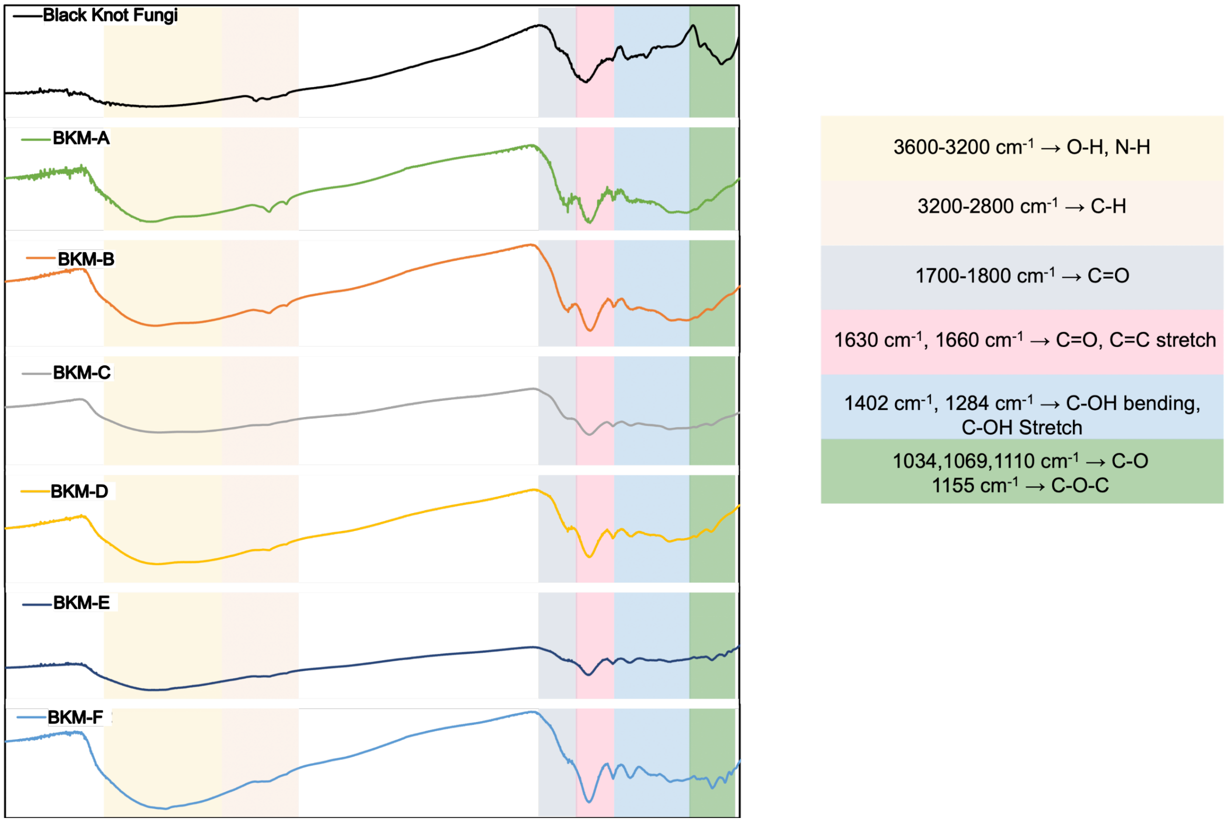

Fig. S13. FTIR of black knot melanin extracted under conditions following Table S7. Analysis done in solid state using a KBr pellet. IR spectrum showcases no significant changes in the chemical structure of the melanin.

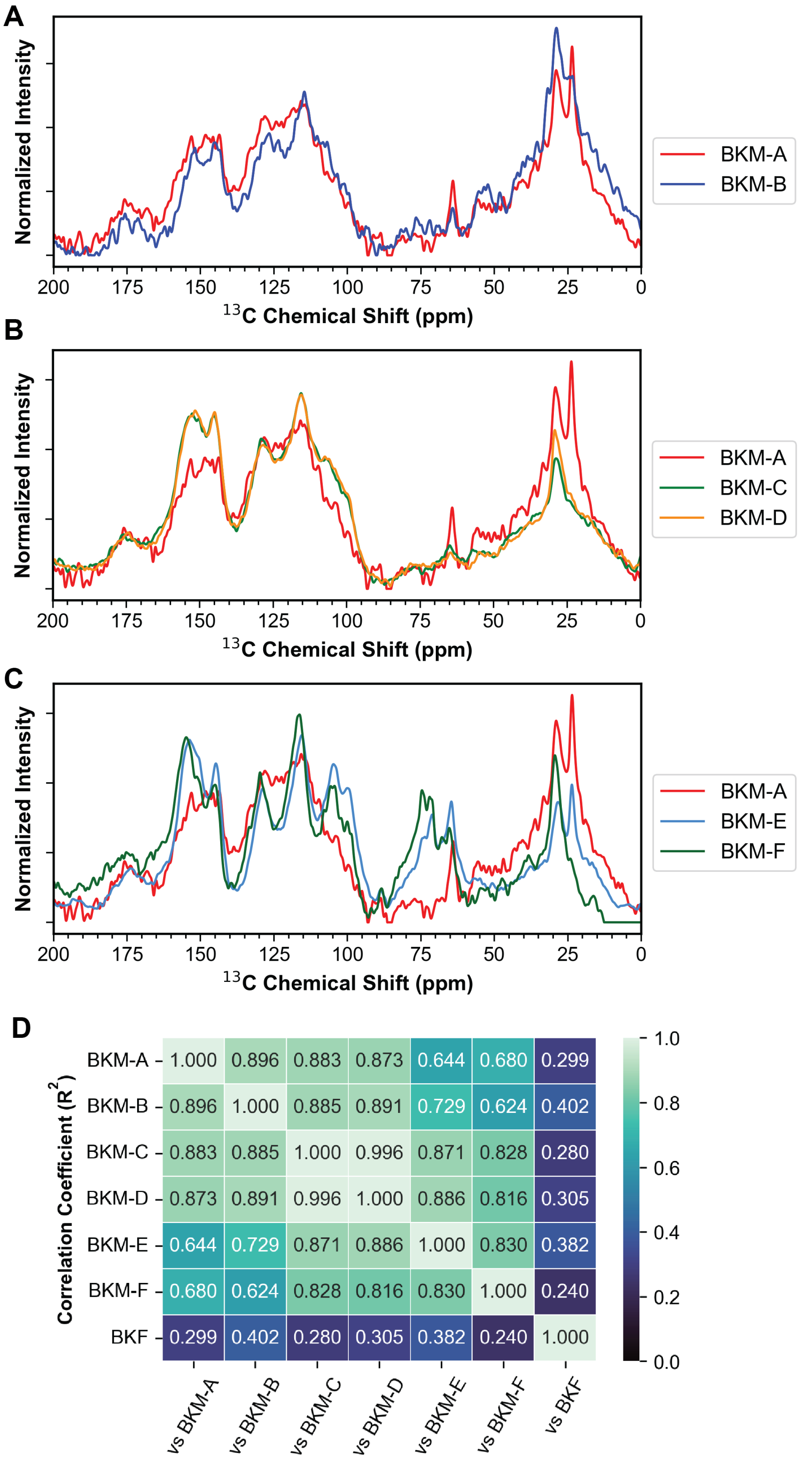

Fig. S14. ^{13}C multi-CP-MAS ssNMR evaluation of alternative extraction techniques (Table S7). (A, B, C) Spectra plotted against the control extraction protocol (BKM-A). (D) Correlation coefficients (R^2) indicating similarities between ^{13}C multi-CPMAS ssNMR spectra of different extraction techniques. Note that when comparing natural allomelanin to itself, the R^2 value will be 1.

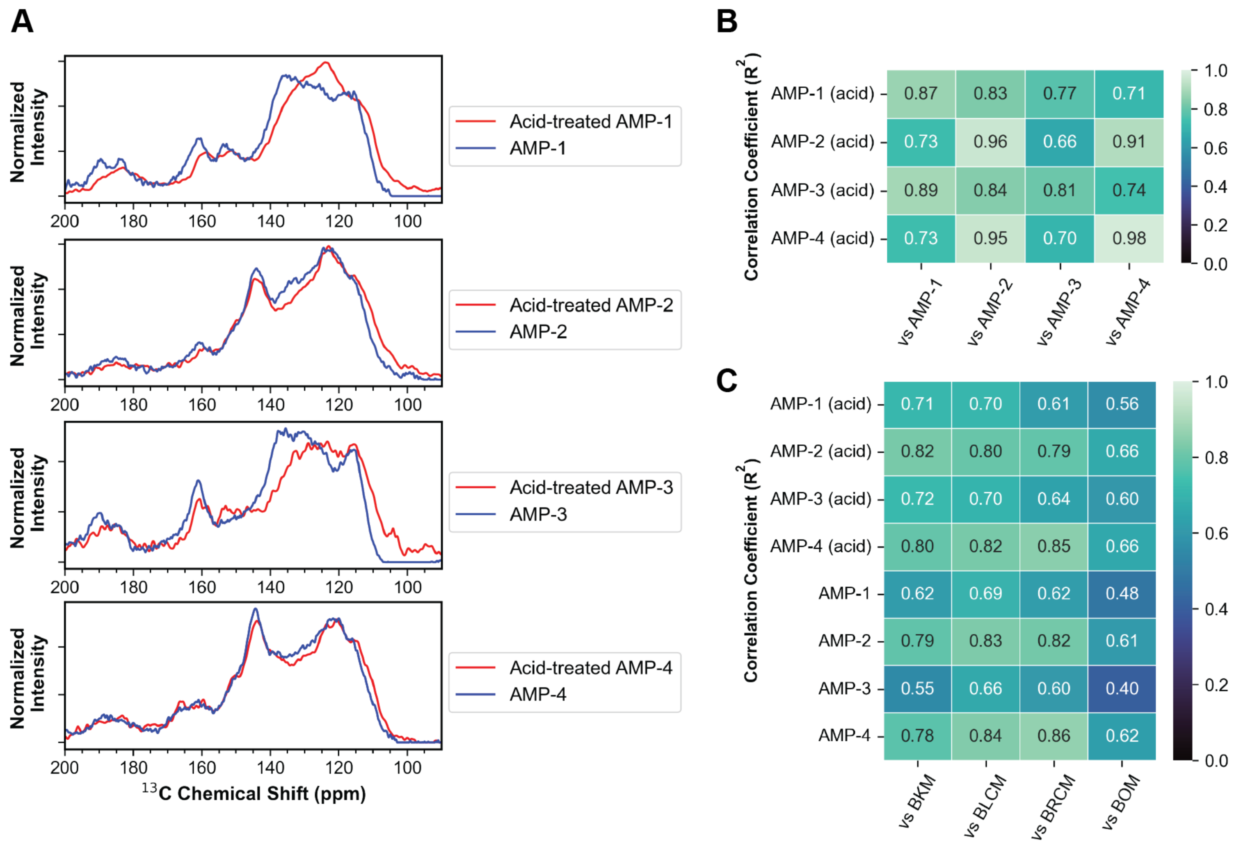

Fig. S15. ^{13}C multi-CPMAS ssNMR for acid-reflux treated synthetic melanin. (A) ^{13}C multi-CPMAS ssNMR spectra of the acid-treated synthetic melanins and their non-treated counterparts (B) Correlation coefficients ($\boldsymbol{R^2}$) indicating similarities between ^{13}C multi-CPMAS ssNMR spectra of acid-treated synthetic melanins and their non-treated counterparts. (C) Correlation coefficients ($\boldsymbol{R^2}$) indicating similarities between ^{13}C multi-CPMAS ssNMR spectra of acid-treated and non-acid treated synthetic melanins and the acid-base extracted natural allomelanins.

Fig. S15. In-depth explanation: The changes to the AMP-1 and AMP-3 spectra originate in the decrease in peak intensities specifically at 161 ppm and 135-140 ppm. The decrease in peak intensity at 161 ppm has been observed in the literature and attributed to C–O–C bonds that lose their intensity upon exposure to heat.(*69*) The decrease in peak intensity between 135-140 ppm is present to some extent in all the synthetic allomelanins and corresponds generally to the broad population of aromatic carbons. Since a decrease in peak intensity at 135-140 ppm was not observed after heat treatment according to previous literature (*69*), this decrease in peak intensity is likely associated with interaction with the acid treatment.

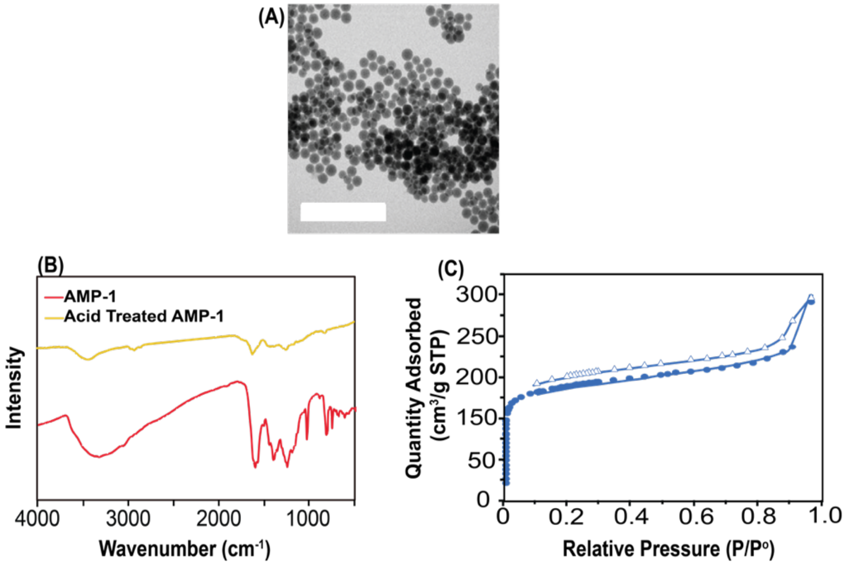

Fig. S16. Characterizing acid-treated AMP-1. A) STEM-TE micrograph (scale bar 1μm), B) FTIR, C) Nitrogen Isotherm.

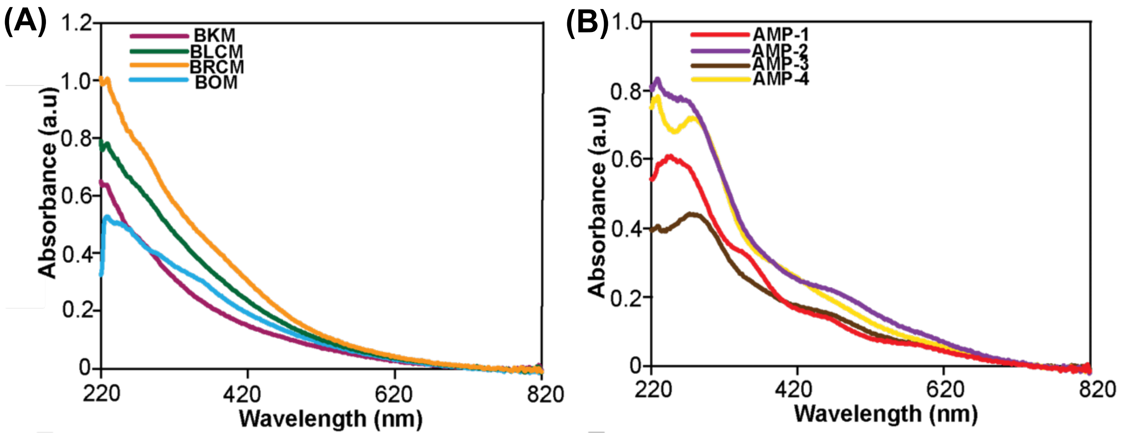

Fig S17. UV-Vis Absorbance for (A) Natural Melanin (B) Synthetic Melanin at 10ug/mL concentration in water

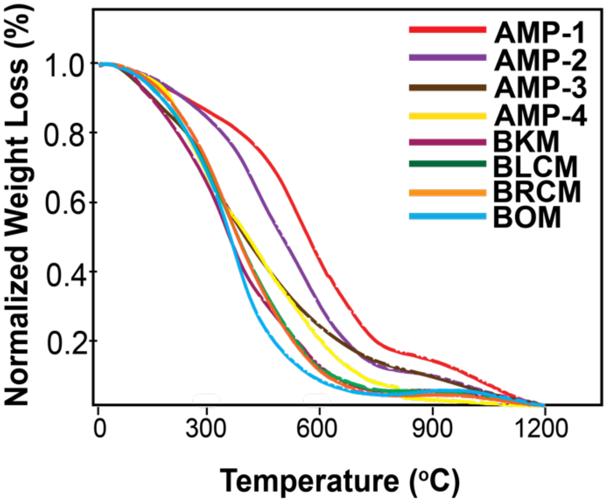

Fig. S18. Thermogravimetric analysis of all extracted and synthetic allomelanin samples.

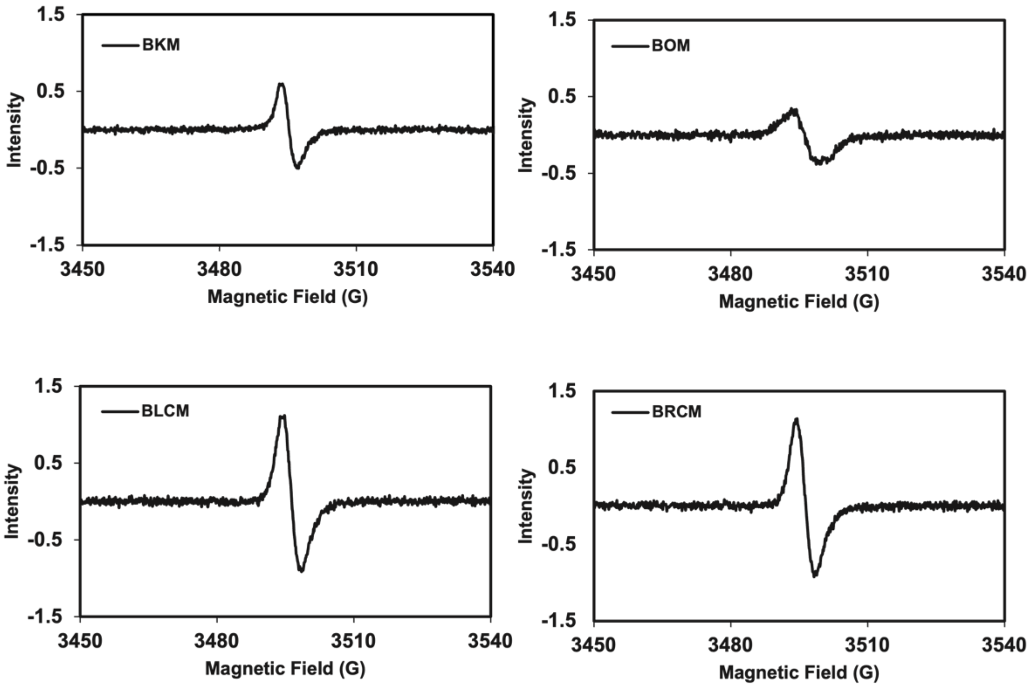

Fig. S19. EPR of natural extracted allomelanin samples.

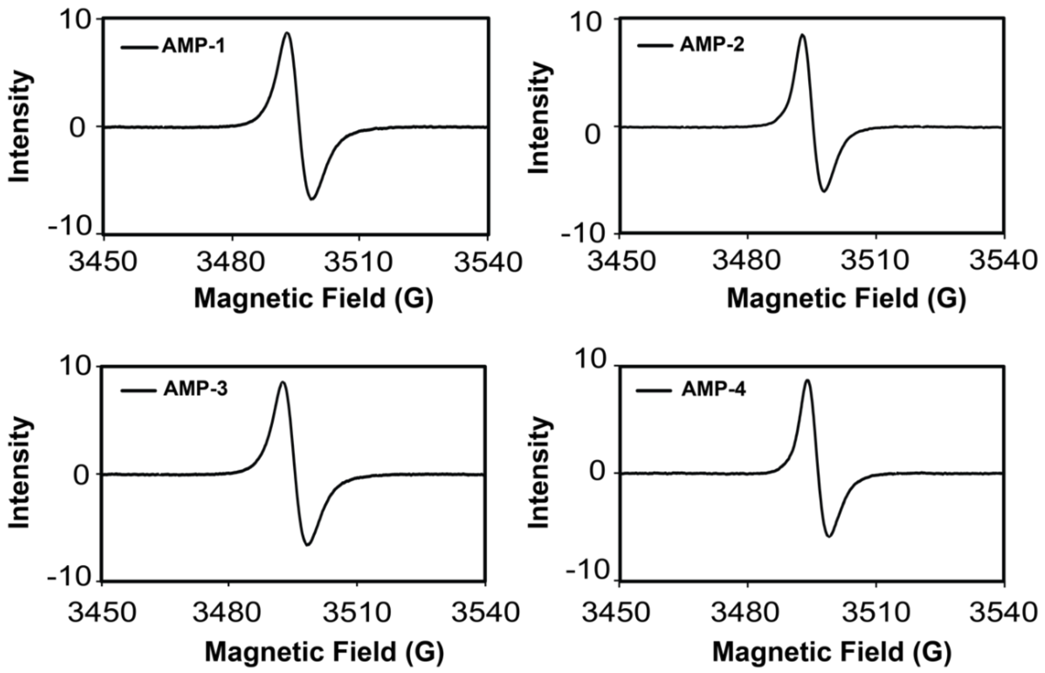

Fig. S20. EPR of synthetic allomelanin.

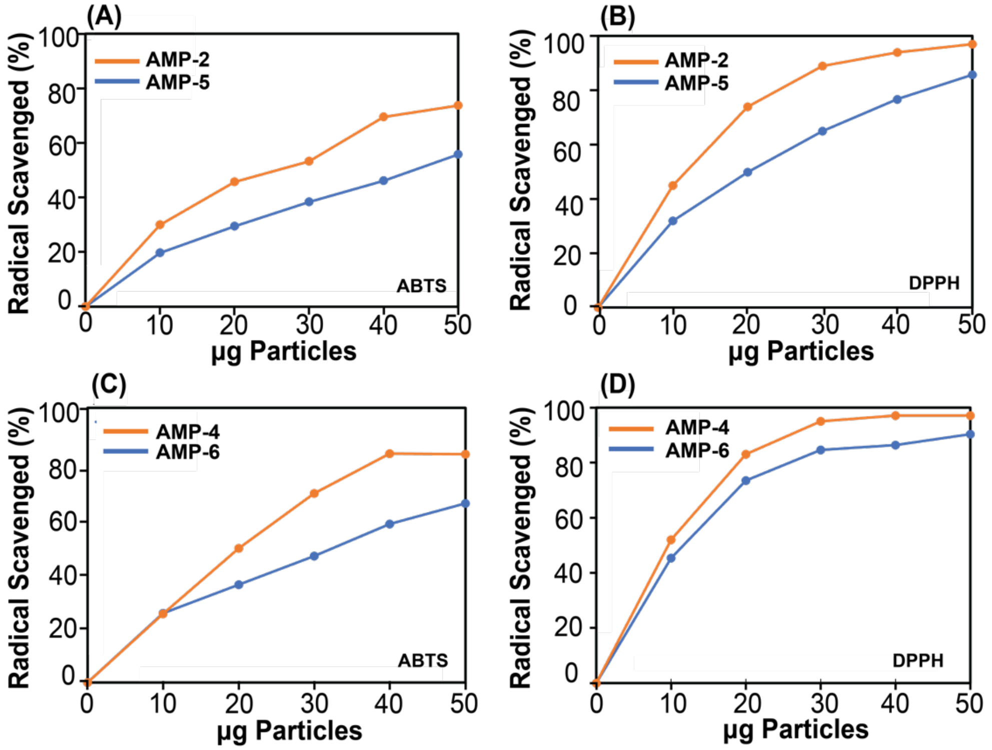

Fig. S21. Radical Scavenging Assays for synthetic allomelanin variants AMP-5 (3.125mM 1,8-DHN, 2.27mM Catechol) A) ABTS assay, B) DPPH assay, and AMP-6 (3.125mM 1,8-DHN, 2.27mM Catechol, 0.2 mM Tannic Acid) C) ABTS assay and D) DPPH assay.

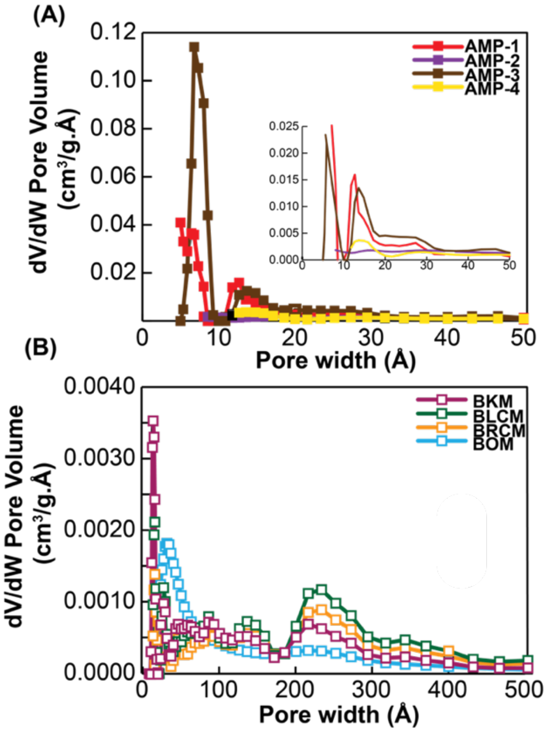

Fig. S22. DFT calculated pore size distribution for A) synthetic allomelanin B) natural extracted allomelanin from Nitrogen isotherms at 77 K.

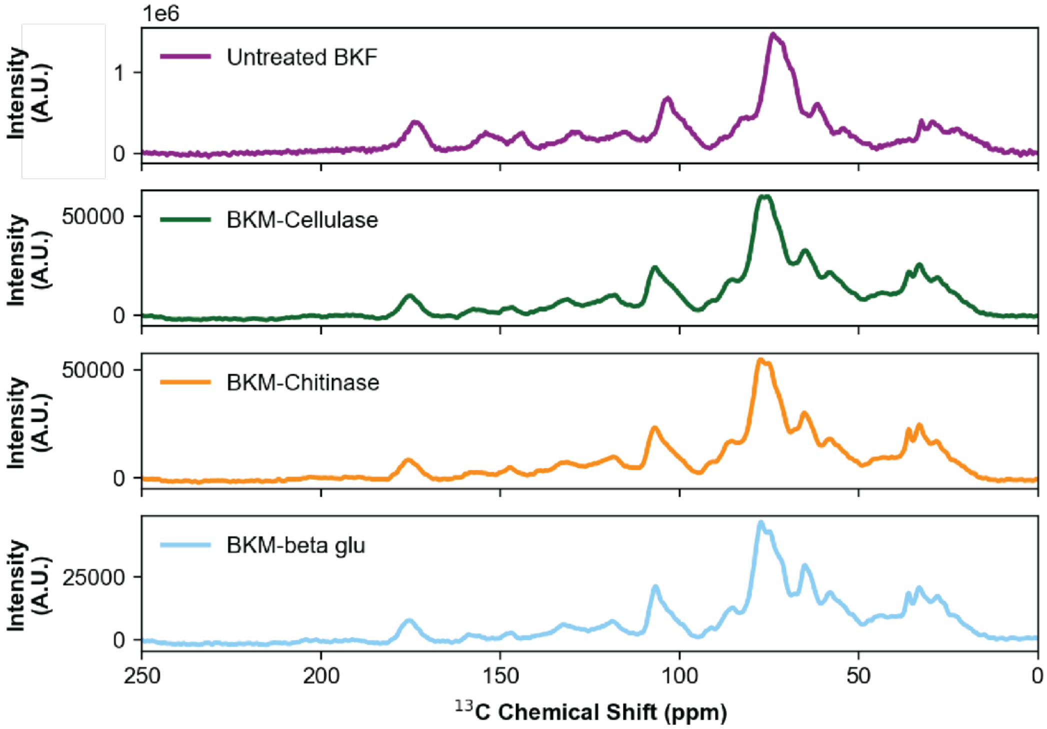

Fig. S23. ^{13}C CPMAS ssNMR spectra of enzymatically extracted melanin from BKF. We observe that there was no decrease in the signals of protein and polysaccharides adhered to melanin compared to the pristine Black Knot Fungi.

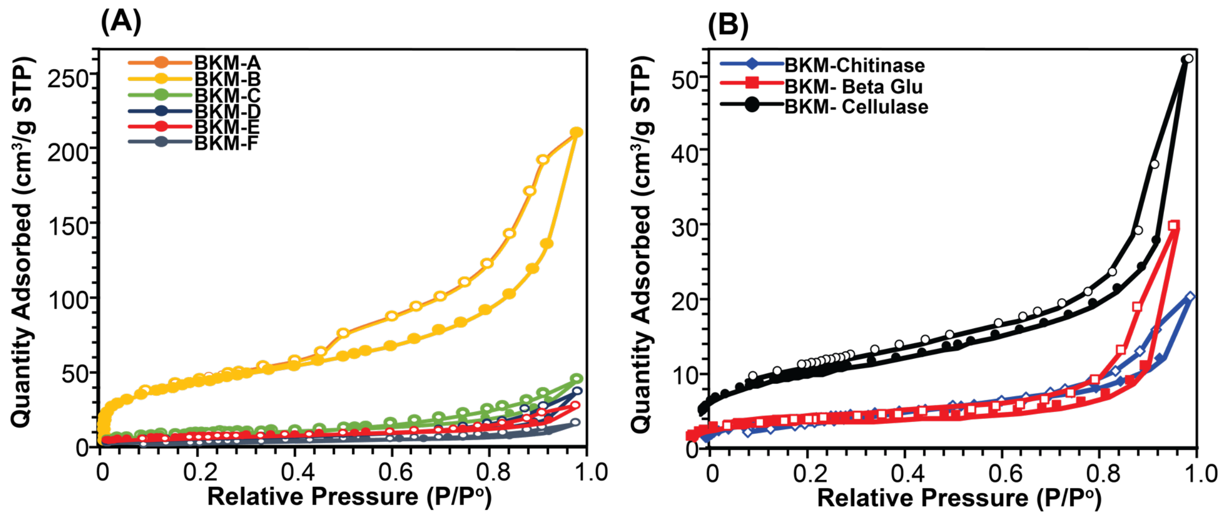

Fig. S24. N_2 Isotherms at 77 K for extracted Black Knot Melanin (BKM) using (A) Milder acid-base conditions as reported in Table S7, (B) Chemoenzymatic extraction. BKM-A and BKM-B exhibit similar isotherm profiles, indicating that the choice of solvent does not significantly influence the porosity of the samples. However, under both milder acid-base extraction conditions and chemoenzymatic conditions, impurities surrounding the melanin are not effectively removed, leading to a reduction in the observed porosity showcased via negligible uptake.

**Natural Source**	**Melanin Location**	**Environment/ Climate**	**Description**
Black Knot Fungi	Black ends of the fungi	Humid Spring	Fungal disease of the prunus genus trees(*1*)
Chaga Mushroom	Brown and black side both have melanin	Cold Climate	Parasitic on birch trees commonly drank as tea to boost the immune system(*2*)
Black Oat	Melanized husks	Cold, Moist Climate	A common oat plant used in immune system supplements(*3*)

Table S1. Location of melanin within the selected sources and the natural environments these plants and fungi are found in.

Sample	Carbon (%)	Oxygen (%)	Nitrogen (%)
**BKF**	65.74	29.93	4.33
**BCM**	70.04	27.90	2.06
**BO**	76.79	19.38	3.83

Table S2. Atomic percentages of different elements as detected by XPS for pristine natural sources.

**Sample**	**Carbon (%)**	**Oxygen (%)**	**Nitrogen (%)**
**BKM**	71.05	28.00	0.95
**BLCM**	71.75	28.25	-
**BRCM**	71.98	28.02	-
**BOM**	79.90	20.10	-

Table S3. Atomic percentages of different elements as detected by XPS for extracted melanin.

**Sample**	**C-C/C=C**	**C-O/C-N**	**C=O**	**O-C=O**	$\pi - \pi *$
AMP-1	284.8	286.3	287.7	289.2	291.2
AMP-2	284.8	286.5	287.6	288.9	291.3
AMP-3	284.8	286.5	287.7	289.2	291.0
AMP-4	284.8	286.4	287.8	289.2	291.5
BKM	284.8	286.29	287.6	289.1	291.3
BLCM	284.8	286.4	287.7	289.1	290.9
BRCM	284.8	286.3	287.3	289.1	291.4
BOM	284.8	286.3	287.6	289.0	290.9

Table S4. Binding energy (eV) of different carbon bonds in both synthetic melanin analogues and natural melanin extracts via XPS. All peaks were charge shifted at 284.8 eV for Carbon.

Sample	C-C/C=C	C-O/C-N	C=O	O-C=O	$\pi - \pi *$
AMP-1	60.24	21.08	9.63	3.61	5.42
AMP-2	68.02	15.64	5.44	4.76	6.12
AMP-3	66.67	18.00	6.67	4.00	4.67
AMP-4	58.82	20.58	12.94	2.35	5.29
BKM	59.52	25.59	5.95	6.54	2.38
BLCM	68.02	16.32	6.80	5.44	3.40
BRCM	64.51	11.61	10.96	9.03	3.87
BOM	70.92	15.60	5.67	7.09	0.70

Table S5. Area percentages of different carbon bonds in synthetic melanin analogues and natural melanin extracts as determined by XPS. The ratio of C–C/C=C to C–O peaks in natural melanin samples (except BKM) is notably lower, likely due to residual covalently bound lipids and reminiscent of chitin and polyscaharides (*15*) that remain after extraction. This results in a dominant C-O band that masks other carbon signals, altering the relative area percentages. This feature is also seen in FTIR and ssNMR data. Additionally, sample-specific impurities contribute to variations in peak intensities, making this XPS data primarily qualitative. Furthermore, differences among the synthetic melanins are expected, as they are co-polymers composed of various monomer species.

Sample	Carbon (%)	Oxygen (%)
**AMP-1**	76.27	23.73
**AMP-2**	70.64	29.36
**AMP-3**	78.90	21.10
**AMP-4**	72.95	27.05

Table S6. Atomic percentages of different elements as detected by XPS for synthetic allomelanin analogues.

Sample ID	Grinding	Boiling	pH of Base	Autoclave	Acid Reflux (pH)	Solvent Wash
BKM-A	Yes	Yes	14	Yes	1.0	EtOH
BKM-B	Yes	Yes	14	Yes	1.0	Me_2CO/MeOH
BKM-C	Yes	No	10	Yes	2.5	EtOH
BKM-D	Yes	No	10	Yes	2.5	Me_2CO/MeOH
BKM-E	Yes	No	10	No	2.5	EtOH
BKM-F	Yes	No	10	No	2.5	Me_2CO/MeOH

Table S7. Acid base extraction of melanin from natural sources at milder modified conditions.

Sample	Free Radical Content Per Mass of Melanin (mol/g)	Gaussian linewidth (mT)	Lorentizian Linewidth (mT)	Corrected g-factor
**AMP-1**	67 ± 5	0.42	0.28	2.0038
**AMP-2**	51 ± 5	0.38	0.2	2.0037
**AMP-3**	29 ± 1	0.36	0.3	2.0038
**AMP-4**	48 ± 0.2	0.38	0.19	2.0037
**BKM**	1.84 ± 0.18	0.27	0.12	2.0039
**BLCM**	2 ± 0.06	0.32	0.15	2.0036
**BRCM**	2.13 ± 0.04	0.34	0.12	2.0036
**BOM**	1.7 ± 0.1	0.45	0.26	2.0037

Table S8. Free Radical content, linewidth, and g-factor values for allomelanin as determined by EPR using a 4-hydroxy TEMPO calibration curve, developed freshly on the day of each measurement.(*65*) The line width of the melanin species is narrower than what is reported in the literature,(*45, 46*) This is attributed to the fact that we ran our samples in a liquid state which rather than a solid state, leading to a more non-aggregated sample, therefore avoiding the freezing effect. The reduced g-factor relative to nitrogenous eumelanin analogues indicates free, stable radicals with a predominantly C–C–centered character in the fungal melanin and its mimics.(*45*)

Sample	Diameter by DLS (nm)	Zeta Potential (mV)	BET Surface Area (m^2/g)	Pore Sizes (nm)	Pore Volume (cm^3/g)
**AMP-1**	140 ± 2	-32.48	800	0.65,1.3,2.7	0.337
**AMP-2**	147 ± 1.3	-46.10	205	1.4,2.7, mesopores	0.192
**AMP-3**	158 ± 1.4	-37.89	450	0.13,1.7,2.8	0.285
**AMP-4**	210 ± 25	-38.69	79	1.4, mesopores	0.195
**BKM**	Aggregates	-47.69	155	1.4, mesopores	0.1563
**BLCM**	Aggregates	-53.39	60	0.5,1.3 mesopores	0.0228
**BRCM**	Aggregates	-57.13	50	0.5,1.5, mesopores	0.0283
**BOM**	Aggregates	-38.34	38	mesopores	0.0101

Table S9. Surface characterization and porosity parameters for synthetic and natural melanin samples.

Sample	BET Area (m^2/g)	Pore Sizes (nm)
**BKM-A**	155	1.4, mesopores
**BKM-B**	147	1.3, mesopores
**BKM-C**	26	0.7, 1.3, mesopores
**BKM-D**	20	0.7, 1.8, mesopores
**BKM-E**	18	0.7, 1.0, mesopores
**BKM-F**	19	0.7, 1.0, mesopores

Table S10. Porosity parameters for black knot melanin (BKM) extracted through milder acid-base conditions as mentioned in Table S7.

Sample	BET Area (m^2/g)	Pore Sizes (nm)
**BKM-Cellulase**	30	1.6,2.9,4.6, + mesopores
**BKM- Beta Glu**	20	1.4 + mesopores
**BKM Chitinase**	15	1.5,2.7, + mesopores

Table S11. Porosity parameters for chemoenzymatically extracted melanin from Black Knot Fungi (BKF).

REFERENCES

1. W. Cao, X. Zhou, N. C. McCallum, Z. Hu, Q. Z. Ni, U. Kapoor, C. M. Heil, K. S. Cay, T. Zand, A. J. Mantanona, A. Jayaraman, A. Dhinojwala, D. D. Deheyn, M. D. Shawkey, M. D. Burkart, J. D. Rinehart, N. C. Gianneschi, Unraveling the structure and function of melanin through synthesis. *J. Am. Chem. Soc.* **143**, 2622–2637 (2021).

2. M. Brenner, V. J. Hearing, The protective role of melanin against UV damage in human skin. *Photochem. Photobiol.* **84**, 539–549 (2008).

3. X. Fu, M. Xie, M. Lu, L. Shi, T. Shi, M. Yu, Characterization of the physicochemical properties, antioxidant activity, and antiproliferative activity of natural melanin from *S. reiliana*. *Sci. Rep.* **12**, 2110 (2022).

4. E. Dadachova, A. Casadevall, Ionizing radiation: How fungi cope, adapt, and exploit with the help of melanin. *Curr. Opin. Microbiol.* **11**, 525–531 (2008).

5. B.-L. L. Seagle, K. A. Rezai, E. M. Gasyna, Y. Kobori, K. A. Rezaei, J. R. Norris Jr., Time-resolved detection of melanin free radicals quenching reactive oxygen species. *J. Am. Chem. Soc.* **127**, 11220–11221 (2005).

6. R. J. B. Cordero, A. Casadevall, Functions of fungal melanin beyond virulence. *Fungal Biol. Rev.* **31**, 99–112 (2017).

7. L. D'Alba, M. D. Shawkey, Melanosomes: Biogenesis, properties, and evolution of an ancient organelle. *Physiol. Rev.* **99**, 1–19 (2019).

8. A. Y. Glagoleva, O. Y. Shoeva, E. K. Khlestkina, Melanin pigment in plants: Current knowledge and future perspectives. *Front. Plant Sci.* **11**, 770 (2020).

9. K. L. Robertson, A. Mostaghim, C. A. Cuomo, C. M. Soto, N. Lebedev, R. F. Bailey, Z. Wang, Adaptation of the black yeast *Wangiella dermatitidis* to ionizing radiation: Molecular and cellular mechanisms. *PLOS ONE* **7**, e48674 (2012).

10. C. Pacelli, R. A. Bryan, S. Onofri, L. Selbmann, I. Shuryak, E. Dadachova, Melanin is effective in protecting fast and slow growing fungi from various types of ionizing radiation. *Environ. Microbiol.* **19**, 1612–1624 (2017).

11. X. Zhou, N. C. McCallum, Z. Hu, W. Cao, K. Gnanasekaran, Y. Feng, J. F. Stoddart, Z. Wang, N. C. Gianneschi, Artificial allomelanin nanoparticles. *ACS Nano* **13**, 10980–10990 (2019).

12. N. C. McCallum, F. A. Son, T. D. Clemons, S. J. Weigand, K. Gnanasekaran, C. Battistella, B. E. Barnes, H. Abeyratne-Perera, Z. E. Siwicka, C. J. Forman, X. Zhou, M. H. Moore, D. A. Savin, S. I. Stupp, Z. Wang, G. J. Vora, B. J. Johnson, O. K. Farha, N. C. Gianneschi, Allomelanin: A biopolymer of intrinsic microporosity. *J. Am. Chem. Soc.* **143**, 4005–4016 (2021).

13. Z. E. Siwicka, F. A. Son, C. Battistella, M. H. Moore, J. Korpanty, N. C. McCallum, Z. Wang, B. J. Johnson, O. K. Farha, N. C. Gianneschi, Synthetic porous melanin. *J. Am. Chem. Soc.* **143**, 3094–3103 (2021).

14. J. D. Nosanchuk, R. E. Stark, A. Casadevall, Fungal melanin: What do we know about structure? *Front. Microbiol.* **6**, 1463 (2015).

15. S. Singla, K. Z. Htut, R. Zhu, A. Davis, J. Ma, Q. Z. Ni, M. D. Burkart, C. Maurer, T. Miyoshi, A. Dhinojwala, Isolation and characterization of allomelanin from pathogenic black knot fungus—A sustainable source of melanin. *ACS Omega* **6**, 35514–35522 (2021).

16. C. W. Wold, C. Kjeldsen, A. Corthay, F. Rise, B. E. Christensen, J. Ø. Duus, K. T. Inngjerdingen, Structural characterization of bioactive heteropolysaccharides from the medicinal fungus *Inonotus obliquus* (Chaga). *Carbohydr. Polym.* **185**, 27–40 (2018).

17. M. Varga, O. Berkesi, Z. Darula, N. V. May, A. Palágyi, Structural characterization of allomelanin from black oat. *Phytochemistry* **130**, 313–320 (2016).

18. W. G. D. Fernando, J. X. Zhang, C. Q. Chen, W. R. Remphrey, A. Schurko, G. R. Klassen, Molecular and morphological characteristics of *Apiosporina morbosa*, the causal agent of black knot in *Prunus* spp. *Can. J. Plant Pathol.* **27**, 364–375 (2005).

19. C. W. Wold, W. H. Gerwick, H. Wangensteen, K. T. Inngjerdingen, Bioactive triterpenoids and water-soluble melanin from *Inonotus obliquus* (Chaga) with immunomodulatory activity. *J. Funct. Foods* **71**, 104025 (2020).

20. A. N. Shikov, O. N. Pozharitskaya, V. G. Makarov, H. Wagner, R. Verpoorte, M. Heinrich, Medicinal plants of the Russian Pharmacopoeia; their history and applications. *J. Ethnopharmacol.* **154**, 481–536 (2014).

21. R. R. Safin, S. R. Mukhametzyanov, V. V. Gubernatorov, Water vacuum-oscillating extraction of Chaga. *IOP Conf. Ser. Mater. Sci. Eng.* **666**, 012086 (2019).

22. S. Roychoudhury, B. Sinha, B. P. Choudhury, N. K. Jha, P. Palit, S. Kundu, S. C. Mandal, A. Kolesarova, M. I. Yousef, J. Ruokolainen, P. Slama, K. K. Kesari, Scavenging properties of plant-derived natural biomolecule para-coumaric acid in the prevention of oxidative stress-induced diseases. *Antioxidants* **10**, 1205 (2021).

23. J.-J. Oh, J. Y. Kim, Y. J. Kim, S. Kim, G.-H. Kim, Utilization of extracellular fungal melanin as an eco-friendly biosorbent for treatment of metal-contaminated effluents. *Chemosphere* **272**, 129884 (2021).

24. J. V. Paulin, J. D. McGettrick, C. F. O. Graeff, A. B. Mostert, Melanin system composition analyzed by XPS depth profiling. *Surf. Interfaces* **24**, 101053 (2021).

25. B. N. Kalaj, Q. Z. Ni, J. J. La Clair, D. D. Deheyn, M. D. Burkart, Chemoenzymatic isolation and characterization of high purity mammalian melanin. *Chembiochem* **23**, e202200021 (2022).

26. A. A. Bell, M. H. Wheeler, Biosynthesis and functions of fungal melanins. *Annu. Rev. Phytopathol.* **24**, 411–451 (1986).

27. F. Solano, Melanins: skin pigments and much more—Types, structural models, biological functions, and formation routes. *New J. Sci.* **2014**, 1–28 (2014).

28. G. H. N. Towers, A. Tse, W. S. G. Maass, Phenolic acids and phenolic glycosides of Gaultheria species. *Phytochemistry* **5**, 677–681 (1966).

29. H. S. Reed, J. Dufrenoy, Catechol aggregates in the vacuoles of cells of zinc deficient plants. *Am. J. Bot.* **29**, 544–551 (1942).

30. I. D. Clarke, J. S. Rogers, A. F. Sievers, H. Hopp, "Tannin Content and Other Characteristics of Native Sumac in Relation to its Value as a Commercial Source of Tannin" (1949); https://doi.org/10.22004/AG.ECON.170384.

31. Y. Wang, G. Pan, T. Huang, T. Zhang, J. Lin, L. Song, G. Zhou, X. Ma, Y. Ge, Y. Xu, C. Yuan, N. Zou, Exogenous tannic acid relieves imidacloprid-induced oxidative stress in tea tree by activating antioxidant responses and the flavonoid biosynthetic pathway. *Ecotoxicol. Environ. Saf.* **266**, 115557 (2023).

32. G. Cárdenas, G. Cabrera, E. Taboada, S. P. Miranda, Chitin characterization by SEM, FTIR, XRD, and ^{13}C cross polarization/mass angle spinning NMR. *J. Appl. Polym. Sci.* **93**, 1876–1885 (2004).

33. M. Xiao, W. Chen, W. Li, J. Zhao, Y. Hong, Y. Nishiyama, T. Miyoshi, M. D. Shawkey, A. Dhinojwala, Elucidation of the hierarchical structure of natural eumelanins. *J. R. Soc. Interface* **15**, 20180045 (2018).

34. R. L. Johnson, K. Schmidt-Rohr, Quantitative solid-state ^{13}C NMR with signal enhancement by multiple cross polarization. *J. Magn. Reson.* **239**, 44–49 (2014).

35. J. Mao, X. Cao, D. C. Olk, W. Chu, K. Schmidt-Rohr, Advanced solid-state NMR spectroscopy of natural organic matter. *Prog. Nucl. Magn. Reson. Spectrosc.* **100**, 17–51 (2017).

36. Y. Garro Linck, G. Martínez Delfa, J. A. Donadelli, E. V. Silletta, M. I. Velasco, M. B. Franzoni, G. A. Monti, C. Smal, R. H. Acosta, Total organic carbon determination by ^{13}C CP/MAS solid-state NMR in unconventional oil/gas source rocks: A case study from the Neuquén and Cuyo basins in Argentina. *Energy Fuels* **38**, 5098–5105 (2024).

37. S. Chatterjee, R. Prados-Rosales, B. Itin, A. Casadevall, R. E. Stark, Solid-state NMR reveals the carbon-based molecular architecture of *Cryptococcus neoformans* fungal eumelanins in the cell wall. *J. Biol. Chem.* **290**, 13779–13790 (2015).

38. K. Forfang, B. Zimmermann, G. Kosa, A. Kohler, V. Shapaval, FTIR spectroscopy for evaluation and monitoring of lipid extraction efficiency for oleaginous fungi. *PLOS ONE* **12**, e0170611 (2017).

39. M. M. Urbaniak, M. Gazińska, K. Rudnicka, P. Płociński, M. Nowak, M. Chmiela, In vitro and in vivo biocompatibility of natural and synthetic *Pseudomonas aeruginosa* pyomelanin for potential biomedical applications. *Int. J. Mol. Sci.* **24**, 7846 (2023).

40. F. Lorquin, F. Ziarelli, A. Amouric, C. Di Giorgio, M. Robin, P. Piccerelle, J. Lorquin, Production and properties of non-cytotoxic pyomelanin by laccase and comparison to bacterial and synthetic pigments. *Sci. Rep.* **11**, 8538 (2021).

41. L. Guo, W. Li, Z. Gu, L. Wang, L. Guo, S. Ma, C. Li, J. Sun, B. Han, J. Chang, Recent advances and progress on melanin: From source to application. *Int. J. Mol. Sci.* **24**, 4360 (2023).

42. I.-E. Pralea, R.-C. Moldovan, A.-M. Petrache, M. Ilieș, S.-C. Hegheș, I. Ielciu, R. Nicoară, M. Moldovan, M. Ene, M. Radu, A. Uifălean, C.-A. Iuga, From extraction to advanced analytical methods: The challenges of melanin analysis. *Int. J. Mol. Sci.* **20**, 3943 (2019).

43. L.-F. Wang, J.-W. Rhim, Isolation and characterization of melanin from black garlic and sepia ink. *LWT* **99**, 17–23 (2019).

44. K.-Y. Ju, Y. Lee, S. Lee, S. B. Park, J.-K. Lee, Bioinspired polymerization of dopamine to generate melanin-like nanoparticles having an excellent free-radical-scavenging property. *Biomacromolecules* **12**, 625–632 (2011).

45. N. E.-A. El-Naggar, S. M. El-Ewasy, Bioproduction, characterization, anticancer and antioxidant activities of extracellular melanin pigment produced by newly isolated microbial cell factories *Streptomyces glaucescens* NEAE-H. *Sci. Rep.* **7**, 42129 (2017).

46. E. S. Jacobson, E. Hove, H. S. Emery, Antioxidant function of melanin in black fungi. *Infect. Immun.* **63**, 4944–4945 (1995).

47. L. Leveau, United colours of the city: A review about urbanisation impact on animal colours. *Austral Ecol.* **46**, 670–679 (2021).

48. L. Hong, J. D. Simon, Current understanding of the binding sites, capacity, affinity, and biological significance of metals in melanin. *J. Phys. Chem. B* **111**, 7938–7947 (2007).

49. S. Deguchi, K. Tsujii, K. Horikoshi, In situ microscopic observation of chitin and fungal cells with chitinous cell walls in hydrothermal conditions. *Sci. Rep.* **5**, 11907 (2015).

50. R. P. Baker, C. Chrissian, R. E. Stark, A. Casadevall, Cryptococcus neoformans melanization incorporates multiple catecholamines to produce polytypic melanin. *J. Biol. Chem.* **298**, 101519 (2022).

51. M. Suthar, L. Dufossé, S. K. Singh, The enigmatic world of fungal melanin: A comprehensive review. *J. Fungi* **9**, 891 (2023).

52. M. Pihet, P. Vandeputte, G. Tronchin, G. Renier, P. Saulnier, S. Georgeault, R. Mallet, D. Chabasse, F. Symoens, J.-P. Bouchara, Melanin is an essential component for the integrity of the cell wall of Aspergillus fumigatus conidia. *BMC Microbiol.* **9**, 177 (2009).

53. N. P. Money, T.-C. Caesar-TonThat, B. Frederick, J. M. Henson, Melanin synthesis is associated with changes in hyphopodial turgor, permeability, and wall rigidity in *Gaeumannomyces graminis var. graminis*. *Fungal Genet. Biol.* **24**, 240–251 (1998).

54. Y. Liu, J. D. Simon, The effect of preparation procedures on the morphology of melanin from the ink sac of *Sepia officinalis*. *Pigment Cell Res.* **16**, 72–80 (2003).

55. D. Heidrich, D. M. Pagani, A. Koehler, K. D. O. Alves, M. L. Scroferneker, Effect of melanin biosynthesis inhibition on the antifungal susceptibility of chromoblastomycosis agents. *Antimicrob. Agents Chemother.* **65**, e0054621 (2021).

56. W. F. Paolo, E. Dadachova, P. Mandal, A. Casadevall, P. J. Szaniszlo, J. D. Nosanchuk, Effects of disrupting the polyketide synthase gene *WdPKS1* in *Wangiella* [*Exophiala*] *dermatitidis* on melanin production and resistance to killing by antifungal compounds, enzymatic degradation, and extremes in temperature. *BMC Microbiol.* **6**, 55 (2006).

57. J. García-Rivera, A. Casadevall, Melanization of *Cryptococcus neoformans* reduces its susceptibility to the antimicrobial effects of silver nitrate. *Med. Mycol.* **39**, 353–357 (2001).

58. J. Kaewmalakul, J. D. Nosanchuk, N. Vanittanakom, S. Youngchim, Melanization and morphological effects on antifungal susceptibility of *Penicillium marneffei*. *Antonie Van Leeuwenhoek* **106**, 1011–1020 (2014).

59. S. Liu, S. Youngchim, D. Zamith-Miranda, J. D. Nosanchuk, Fungal melanin and the mammalian immune system. *J. Fungi* **7**, 264 (2021).

60. A. Rella, A. M. Farnoud, M. Del Poeta, Plasma membrane lipids and their role in fungal virulence. *Prog. Lipid Res.* **61**, 63–72 (2016).

61. D. N. Moses, M. A. Mattoni, N. L. Slack, J. H. Waite, F. W. Zok, Role of melanin in mechanical properties of *Glycera jaws*. *Acta Biomater.* **2**, 521–530 (2006).

62. Y. Wang, Z. Wang, P. Ma, H. Bai, W. Dong, Y. Xie, M. Chen, Strong nanocomposite reinforcement effects in poly(vinyl alcohol) with melanin nanoparticles. *RSC Adv.* **5**, 72691–72698 (2015).

63. R. Campana, F. Fanelli, M. Sisti, Role of melanin in the black yeast fungi *Aureobasidium pullulans* and *Zalaria obscura* in promoting tolerance to environmental stresses and to antimicrobial compounds. *Fungal Biol.* **126**, 817–825 (2022).

64. W. Cao, A. J. Mantanona, H. Mao, N. C. McCallum, Y. Jiao, C. Battistella, V. Caponetti, N. Zang, M. P. Thompson, M. Montalti, J. F. Stoddart, M. R. Wasielewski, J. D. Rinehart, N. C. Gianneschi, Radical-enriched artificial melanin. *Chem. Mater.* **32**, 5759–5767 (2020).

65. S. Stoll, A. Schweiger, EasySpin, a comprehensive software package for spectral simulation and analysis in EPR. *J. Magn. Reson.* **178**, 42–55 (2006).

66. A. Zharkikh, 2020.07.16_12.39.27_IMG_8265; https://openverse.org/image/d750b4cf-c130-4bef-9251-3abddea918fe?q=black+knot+fungus&p=4.

67. Rasbak, *Avena sativa* Black Oat (2012); https://commons.wikimedia.org/wiki/File:Avena_sativa_black_oat,_zwarte_haver_(1).jpg#filehistory.

68. T. Montgomery, *Inonotus obliquus*, the medicinal chaga mushroom, Connecticut River Watershed (Vermont, USA, 2015); https://commons.wikimedia.org/wiki/File:Chaga-Tad4.jpg.

69. J. Pukalski, K. Mokrzyński, M. Chyc, M. J. Potrzebowski, T. Makowski, M. Dulski, D. Latowski, Synthesis and characterization of allomelanin model from 1,8-dihydroxynaphthalene autooxidation. *Sci. Rep.* **15**, 567 (2025).
